# Increasing tree size across Amazonia

**DOI:** 10.1038/s41477-025-02097-4

**Published:** 2025-09-25

**Authors:** Adriane Esquivel-Muelbert, Rebecca Banbury Morgan, Roel Brienen, Emanuel Gloor, Simon L. Lewis, Kyle G. Dexter, Everton Almeida, Edmar Almeida de Oliveira, Esteban Álvarez-Dávila, Atila Alves de Oliveira, Ana Andrade, Simone Aparecida Vieira, Luiz Aragão, Alejandro Araujo-Murakami, Eric Arets, Luzmila Arroyo, Gerardo Aymard-Corredor, Olaf Banki, Plinio Barbosa de Camargo, Jorcely Barroso, Lilian Blanc, Foster Brown, José Luís Camargo, Wendeson Castro, Victor Chama Moscoso, Jérôme Chave, Ezequiel Chavez, James Comiskey, Antônio Carlos Lola da Costa, Jhon del Aguila Pasquel, Géraldine Derroire, Anthony Di Fiore, Sophie Fauset, Ted R. Feldpausch, Gerardo Flores Llampazo, Rene Guillen Villaroel, Rafael Herrera, Niro Higuchi, Eurídice Honorio Coronado, Isau Huamantupa-Chuquimaco, Walter Huaraca Huasco, Eliana Jimenez, Timothy Killeen, Susan Laurance, William Laurance, Aurora Levesley, Gabriela Lopez-Gonzalez, Yadvinder Malhi, Beatriz Marimon, Ben Hur Marimon Junior, Simone Matias de Almeida Reis, Casimiro Mendoza Bautista, Irina Mendoza Polo, Abel Monteagudo-Mendoza, Paulo Sérgio Morandi, Adriano Nogueira Lima, Percy Núñez Vargas, Nadir Pallqui Camacho, Alexander Parada Gutierrez, Julie Peacock, Maria Cristina Peñuela-Mora, Georgia Pickavance, John Pipoly, Nigel Pitman, Adriana Prieto, Carlos Quesada, Freddy Ramirez Arevalo, Maxime Réjou-Méchain, Zorayda Restrepo Correa, Rocio Rojas, Lily Rodriguez Bayona, Anand Roopsind, Rafael Salomão, Natalino Silva, Javier Silva Espejo, Marcos Silveira, Juliana Stropp, Joey Talbot, Hans ter Steege, John Terborgh, Raquel Thomas, Luis Valenzuela Gamarra, Peter van der Hout, Rodolfo Vasquez Martinez, Ima Célia Guimarães Vieira, Emilio Vilanova, Roderick Zagt, Timothy R. Baker, Oliver L. Phillips

**Affiliations:** 1 School of Geography, Earth and Environmental Sciences, University of Birmingham, Birmingham, UK; 2 Birmingham Institute of Forest Research (BIFoR), Stafford, UK; 3 Department of Plant Sciences, University of Cambridge, Cambridge, UK; 4 School of Geography, University of Leeds, Leeds, UK; 5 School of Biological Sciences, University of Bristol, Bristol, UK; 6 Department of Geography, University College London, London, UK; 7 University of Turin, Turin, Italy; 8 University of Edinburgh, Edinburgh, UK; 9 Royal Botanic Garden, Edinburgh, UK; 10 Postgraduate Program in Forest Science, Technology and Innovation - Universidade Federal do Oeste do Pará, Santarém, Brazil; 11 Universidade do Estado de Mato Grosso, Cáceres, Brazil; 12 13Fundácion ConVida, Medellín, Colombia; 13 Projeto TEAM, Instituto Nacional de Pesquisas da Amazônia, Manaus, Brazil; 14 Instituto Nacional de Pesquisas da Amazônia, Manaus, Brazil; 15 Universidade Estadual de Campinas, Campinas, Brazil; 16 National Institute for Space Research (INPE), São José dos Campos, Brazil; 17 National Institute for Space Research (INPE), Santa Cruz de la Sierra, Bolivia; 18 Wageningen University and Research, Wageningen, Netherlands; 19 Herbario Universitario (PORT), Guanare, Venezuela; 20 Herbario Universitario (PORT), Bogotá, Colombia; 21 Naturalis Biodiversity Center, Leiden, Netherlands; 22 Centro de Energia Nuclear na Agricultura, Universidade de São Paulo, Piracicaba, Brazil; 23 Universidade Federal do Acre, Rio Branco, Brazil; 24 CIRAD, UPR Forêts et Sociétés, University of Montpellier, Montpellier, France; 25 Woods Hole Research Center, Falmouth, MA USA; 26 CIRAD, UMR EcoFoG (AgroParistech, CNRS, INRAE, Université des Antilles, Université de la Guyane), Oxapampa, Peru; 27 Universidad Nacional de San Antonio Abad del Cusco, Cusco, Peru; 28 CRBE–CNRS, Université de Toulouse, Toulouse, France; 29 Museu Noel Kempff, Santa Cruz de la Sierra, Bolivia; 30 National Park Service, Washington, DC USA; 31 Universidade Federal do Para, Belém, Brazil; 32 Instituto de Investigaciones de la Amazonia Peruana, Iquitos, Peru; 33 Universidad Nacional de la Amazonia Peruana, Iquitos, Peru; 34 CIRAD, UMR EcoFoG (AgroParistech, CNRS, INRAE, Université des Antilles, Université de la Guyane), Kourou, French Guiana; 35 University of Texas at Austin, Austin, TX USA; 36 Universidad San Francisco de Quito, Quito, Ecuador; 37 University of Plymouth, Plymouth, UK; 38 University of Exeter, Exeter, UK; 39 Independent consultant, Santa Cruz de la Sierra, Bolivia; 40 Instituto Venezolano de Investigaciones Científicas (IVIC), Parroquia Macarao, Venezuela; 41 Universidad Nacional Amazónica de Madre de Dios, Puerto Maldonado, Peru; 42 Department of Ecology and Environmental Science, Umeå University, Umeå, Sweden; 43 Environmental Change Institute, School of Geography and the Environment, University of Oxford, Oxford, UK; 44 Universidad Nacional de Colombia, Bogotá, Colombia; 45 Missouri Botanical Garden, St. Louis, MO USA; 46 James Cook University, Townsville, Queensland Australia; 47 University of Oxford, Oxford, UK; 48 Universidad Mayor de San Simón, Cochabamba, Bolivia; 49 Jardín Botánico de Medellín, Medellín, Colombia; 50 Universidad Regional Amazónica Ikiam, Tena, Ecuador; 51 Florida Atlantic University, Boca Raton, FL USA; 52 Field Museum of Natural History, Chicago, IL USA; 53 AMAP - IRD, Montpellier, France; 54 Fundación Con Vida & Corporacion COL-TREE, Medellín, Colombia; 55 Centro de Conservación, Investigación y Manejo de Areas Naturales, San Antonio, Peru; 56 Iwokrama International Centre for Rainforest Conservation and Development, Georgetown, Guyana; 57 Museu Paraense Emilio Goeldi, Belém, Brazil; 58 Serviço Florestal Brasileiro, Santarém, Brazil; 59 Universidad de La Serena, La Serena, Chile; 60 Instituto de Ecología y Biodiversidad (IEB), Santiago, Chile; 61 Instituto de Ecología y Biodiversidad (IEB), Trier, Germany; 62 Utrecht University, Utrecht, Netherlands; 63 Department of Biology, University of Florida, Gainesville, FL USA; 64 School of Science and Engineering, James Cook University, Townsville, Queensland Australia; 65 Form International, Zwolle, Netherlands; 66 Universidad de Los Andes, Mérida, Venezuela

**Keywords:** Forest ecology, Climate-change ecology, Tropical ecology

## Abstract

Climate change and increasing availability of resources such as carbon dioxide are modifying forest functioning worldwide, but the effects of these changes on forest structure are unclear. As additional resources become available, for example, through CO_2_ fertilization or nitrogen deposition, large trees, with greater access to light, may be expected to gain further advantages. Conversely, smaller light-suppressed trees might benefit more if their light compensation point changes, while bigger trees may be the most negatively impacted by increasing heat and drought. We assessed recent changes in the structure of Earth’s largest tropical forest by analysing 30 years of Amazonian tree records across 188 mature forest plots. We find that, at a stand level, trees have become larger over time, with mean tree basal area increasing by 3.3% per decade (95% CI 2.4; 4.1). Larger trees have increased in both number and size, yet we observed similar rates of relative size gain in large and small trees. This evidence is consistent with a resource-driven boost for larger trees but also a reduction in suppression among smaller trees. These results, especially the persistence and consistency of tree size increases across Amazonian forest plots, communities and regions, indicate that any negative impacts of climate change on forests and large trees here have so far been mitigated by the positive effects of increased resources.

## Abstract (web summary)

A global research network monitoring the Amazon for 30 years reports in this study that tree size increased by 3% each decade.

## Main

Forests worldwide are a key component of terrestrial carbon dynamics. While land-use change in the tropics has driven a large net carbon flux to the atmosphere^1^, research in remaining mature tropical forests has revealed substantial and persistent increases in biomass and an associated carbon sink^2–4^. Widespread changes in biomass productivity and mortality are also occurring across tropical forests, with at least some of these changes likely driven by increased resource availability from elevated atmospheric CO_2_ concentrations or nitrogen deposition, and climatic stress with hotter temperatures and more intense and frequent drought and storms^2,5–7^. These drivers can be at times opposing forces, as while greater resource availability stimulates plant growth^8,9^, climate stressors can lead to lower productivity and increase tree mortality rates^10^. The relative contribution of each of these drivers and their net impact on forest structure is poorly understood. To date, the aggregate changes in mature tropical forest biomass have not been interrogated in terms of shifts within forests in size-class dominance and biomass contributions.

**Fig. 1:**
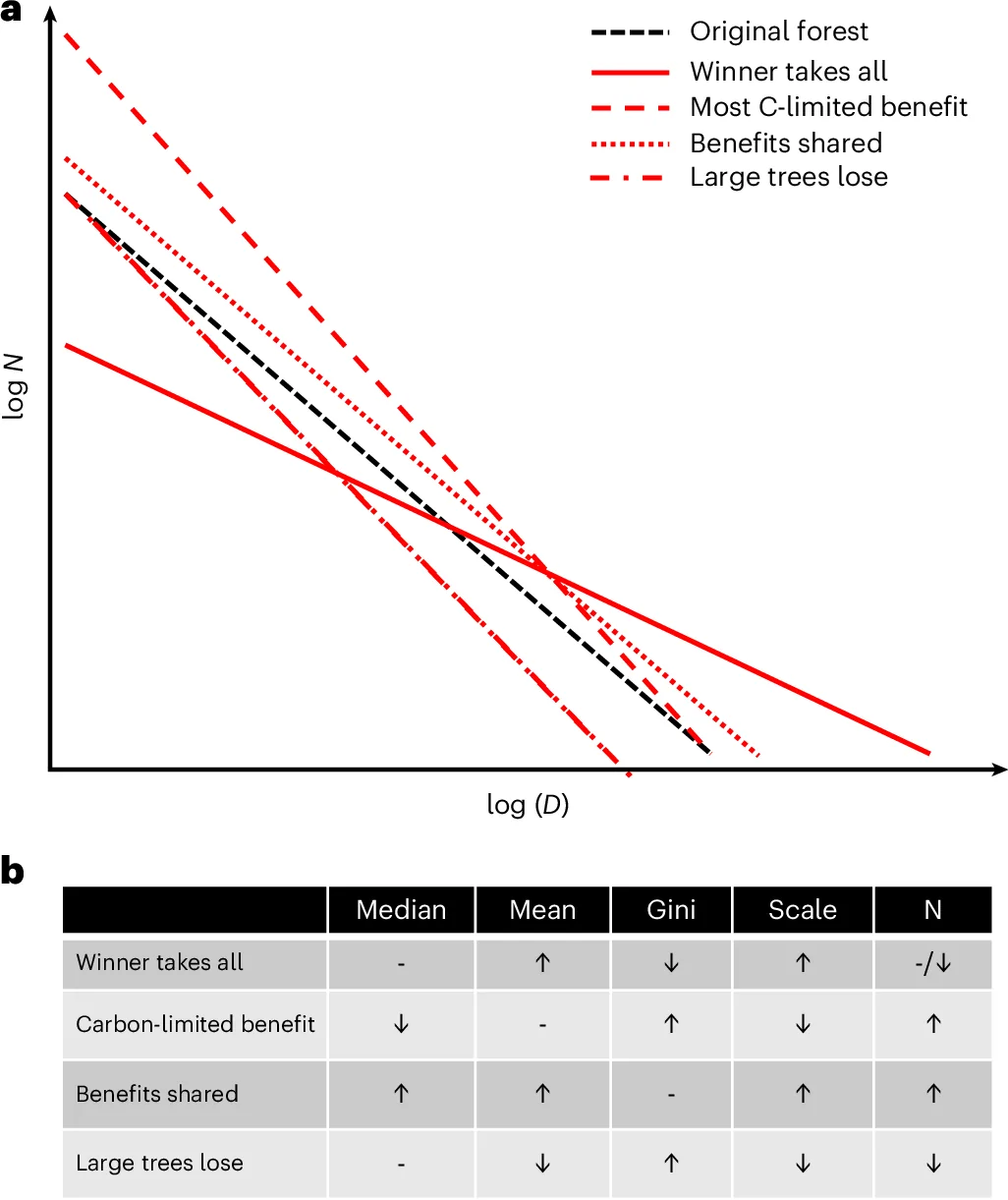
Potential impacts of growth stimulation and climate change on forest structure. **a**, Expected change in tree size distribution under different hypotheses. *D*, diameter at breast height. **b**, Direction of anticipated changes in key tree size descriptors compared to the original forest. Winners-take-all hypothesis: If increase in resources benefits the largest trees, asymmetric competition for light will increase, leading to greater light suppression in the understorey. This will increase the mean tree size but will not affect the median size, potentially decrease stem numbers (*N*), increase the scale parameter (scale) and raise the Gini coefficient. Carbon-limited benefit hypothesis: If CO_2_ stimulates growth in understorey trees, improving their carbon balance, smaller trees will grow more, increasing recruitment in smaller size classes. This will raise stem numbers, decrease median tree size (with little effect on the mean), increase the Gini coefficient and decrease the scale parameter. Shared benefits hypothesis: If increase in resources benefits all trees equally, we expect an increase in mean and median tree sizes and number of stems, a larger scale parameter and no change in the Gini coefficient. Large trees lose hypothesis: If increasing heat, drought, lightning or wind disproportionately impact the mortality of large trees, the mean tree size would decrease, median size would remain unchanged, stem numbers would decline and the scale parameter would lower, with lower inequality (greater Gini coefficient).

The effects of higher resources—either via CO_2_ fertilization or nitrogen deposition—on forest structure are unclear. Some ecological theory predicts a winners-take-all response to the increase in resources, where larger trees obtain disproportionate amounts of resources, outcompeting smaller trees^11–13^. Large trees are—almost by definition—stronger competitors in forests^12,13^. Tree size provides such an advantage that, to reach the canopy, trees invest large amounts of carbon in vertical growth^14^. Greater access to light allows large trees to dominate light capture and thus accumulate more biomass^15^. This boosts their relative fitness by making light unavailable to small trees^12^. As a result, competition between trees is mostly size asymmetric^16^, with larger trees able to exploit greater amounts of resources. Larger trees are also expected to dominate below-ground as investments in foliage reflect larger investments in roots^17^. The more resource-rich the area, the greater the advantage of large trees, either due to size scaling with the capacity to use resources^18^ or because when other resources are not limiting, competition for light becomes even more important^11,19,20^. Following this logic, at higher resource levels we would anticipate a winners-take-all response, where the largest trees are able to acquire a disproportionate amount of the increase in resources. As large trees have high maintenance costs, an increase in resource availability would offer substantial growth advantages. This would further increase light suppression in the understory causing a reduction in growth and potentially an increase in mortality of small trees^14^. Consequently, the structure of the size-class distribution is expected to shift, with more trees observed within the largest size classes and an increase in mean tree size across the forest (Fig. 1).

Alternatively, additional resources could favour the most suppressed trees, facilitating increased growth and survival rates in smaller size classes^8,21,22^. In tropical forests light limitation in the understorey is so strong that understorey trees live close to their light compensation point, that is, on the edge between positive and negative carbon balance^23–25^. Therefore, a small increase in CO_2_ may make a large relative difference to net carbon balance by reducing photorespiration and stimulating photosynthesis, so that the growth of understorey trees increases and, potentially, some trees that would otherwise have died survive^21,26^. If these effects are important, higher CO_2_ levels would weaken the effects of asymmetric competition for light leading to a carbon-limited benefit response (Fig. 1). If this is the case, we expect a greater number of trees within smaller size classes and changes in size on a relative basis to be greater in smaller stems. These potential responses are not necessarily mutually exclusive. Through a combination of the above processes, an increase in resources can have similar impacts across trees regardless of their size leading to a benefits-shared response (Fig. 1).

In parallel to the increase in resource availability, climate-related drivers of change, such as drought, lightning, fire and windthrow are increasing in frequency^27^. Overall, these changes are expected to decrease productivity and increase tree mortality rates, in contrast to the effects of increased resources^5,27,28^. These impacts are expected to be greatest for large trees, which tend to be most vulnerable to these climate drivers^29,30^, and may result in declines in the size and frequency of large trees. Under this scenario, where large trees lose, we expect a redistribution of basal area (BA) towards smaller-size classes, resulting in biomass stocks being increasingly concentrated in small- and medium-sized trees. Whether the structure of natural tree communities in Amazonia has been responding to increase in resources and changing climate in accordance with any of these expectations remains untested.

In this Article, we assess the changes in tree size structure in the Amazon over three decades. A widespread, long-term dataset of mature tropical forest plots is interrogated for structural change, and this information is used to help understand the potential influence of ongoing environmental change on forest structure. Specifically, we test the winners-take-all, carbon-limited-benefit, benefits-shared and large-trees-lose hypotheses by analysing changes in simple size structure parameters (Fig. 1) including mean and median tree size, the size frequency distribution of trees within plots and the distribution of area occupied by individual trees in a plot measured by the Gini coefficient.

## Results

**Fig. 2:**
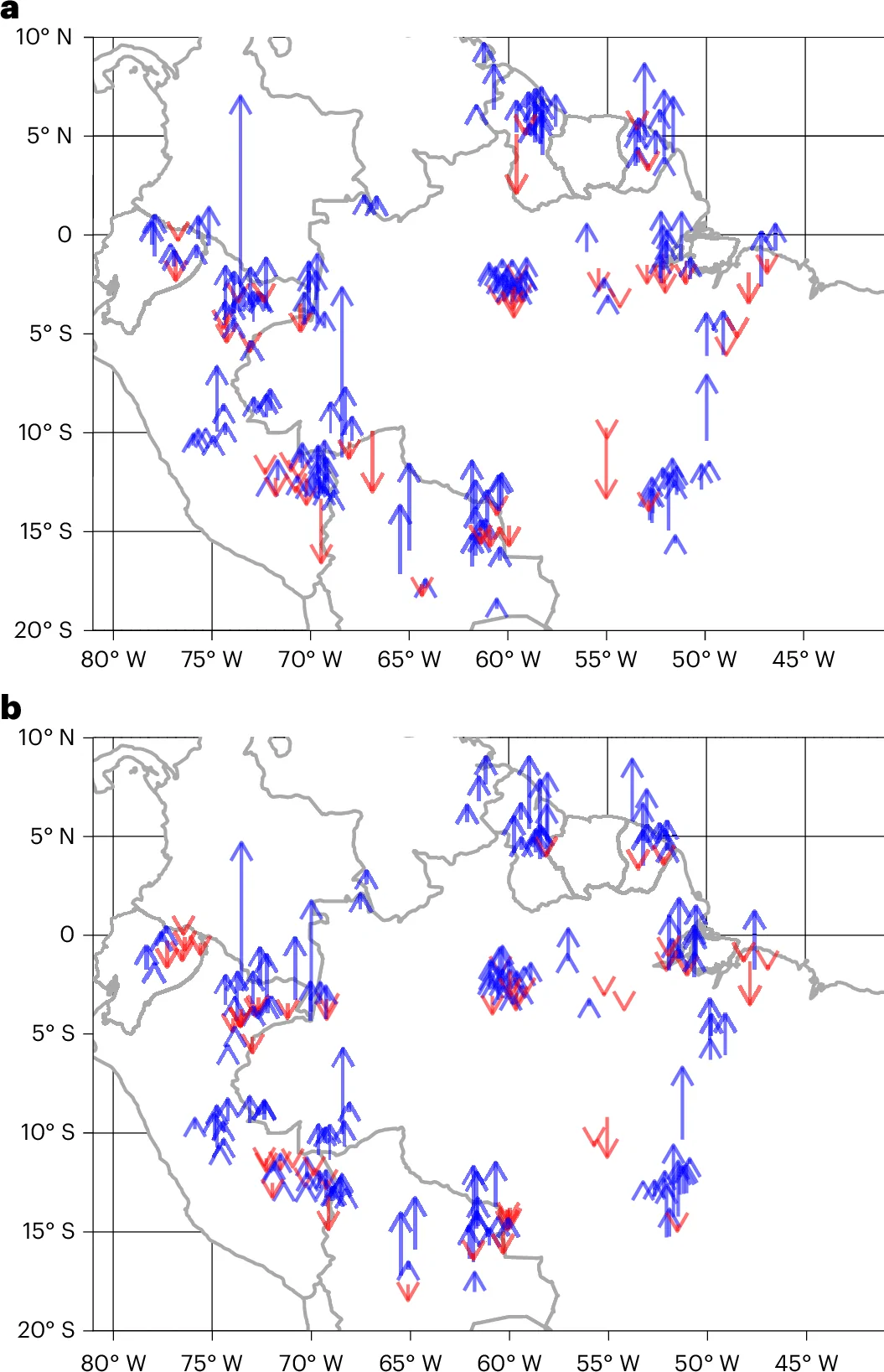
Spatial trends of mean tree size and the scale parameter across Amazonian forests. **a**,**b**, Distribution of annual trends of mean tree BA (**a**) and scale parameter (**b**) per inventory plot across Amazonia. Trends represent the slope of a linear regression fit to mean tree size and scale parameter within each inventory plot. These vary from −7.1 × 10^−4^ to 2.2 × 10^−3^ m^2^ yr^−1^ and −1.1 to 3.7 yr^−1^ for mean tree size and scale parameter, respectively. Mean tree size changed on average by 1.45 × 10^−4^ m^2^ yr^−1^ across all plots, 3.3% gain per decade compared to initial mean size of 4.78 × 10^−2^ m^2^. The average change in scale parameter was 0.4 yr^−1^, 3.8% per decade compared to initial mean scale parameter of 103. Arrows show the magnitude and direction of trends at each plot location, with blue arrows showing increasing trends and red arrows showing declining trends.

**Fig. 3:**
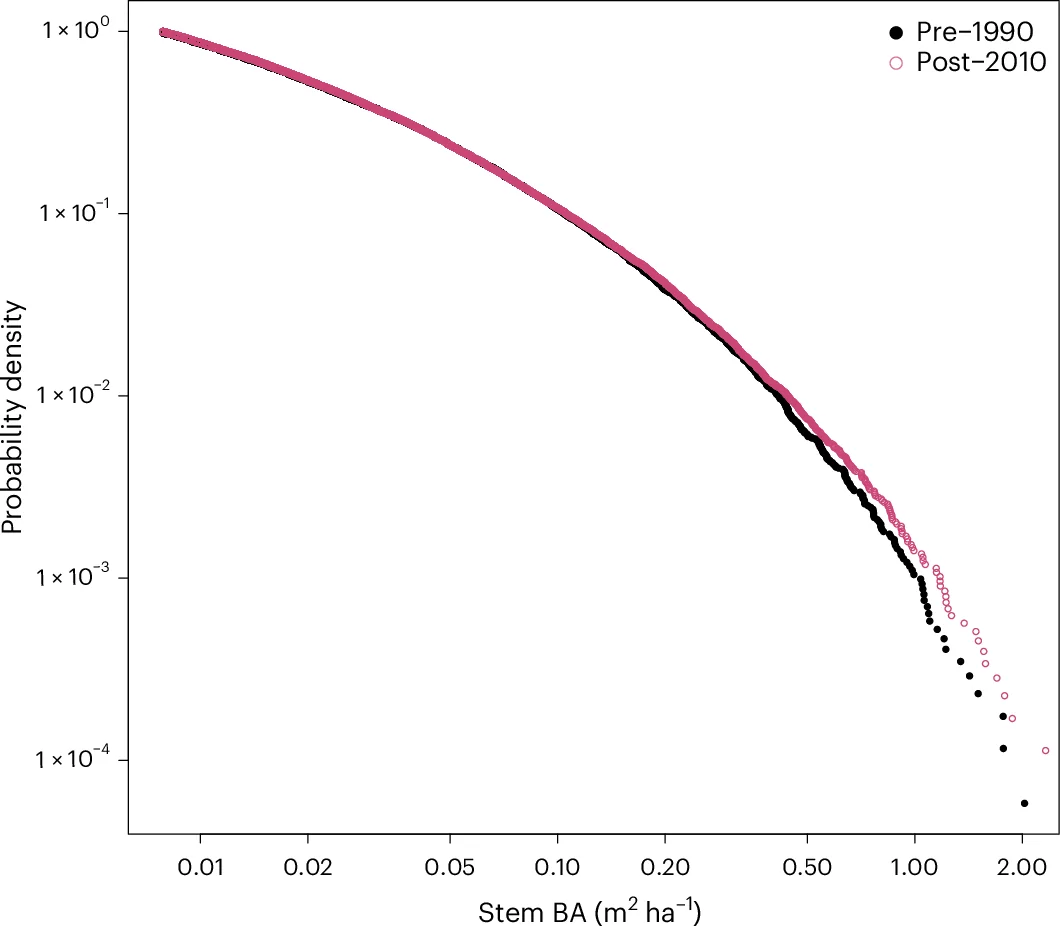
Changes in stem BA distribution between 1990 and 2010. Data are plotted for 30 ha of forest across 22 plots, all censused before 1990 and after 2010, and illustrate an increase in the frequencies and size (shown by the stem BA) of the largest stems.

**Fig. 4:**
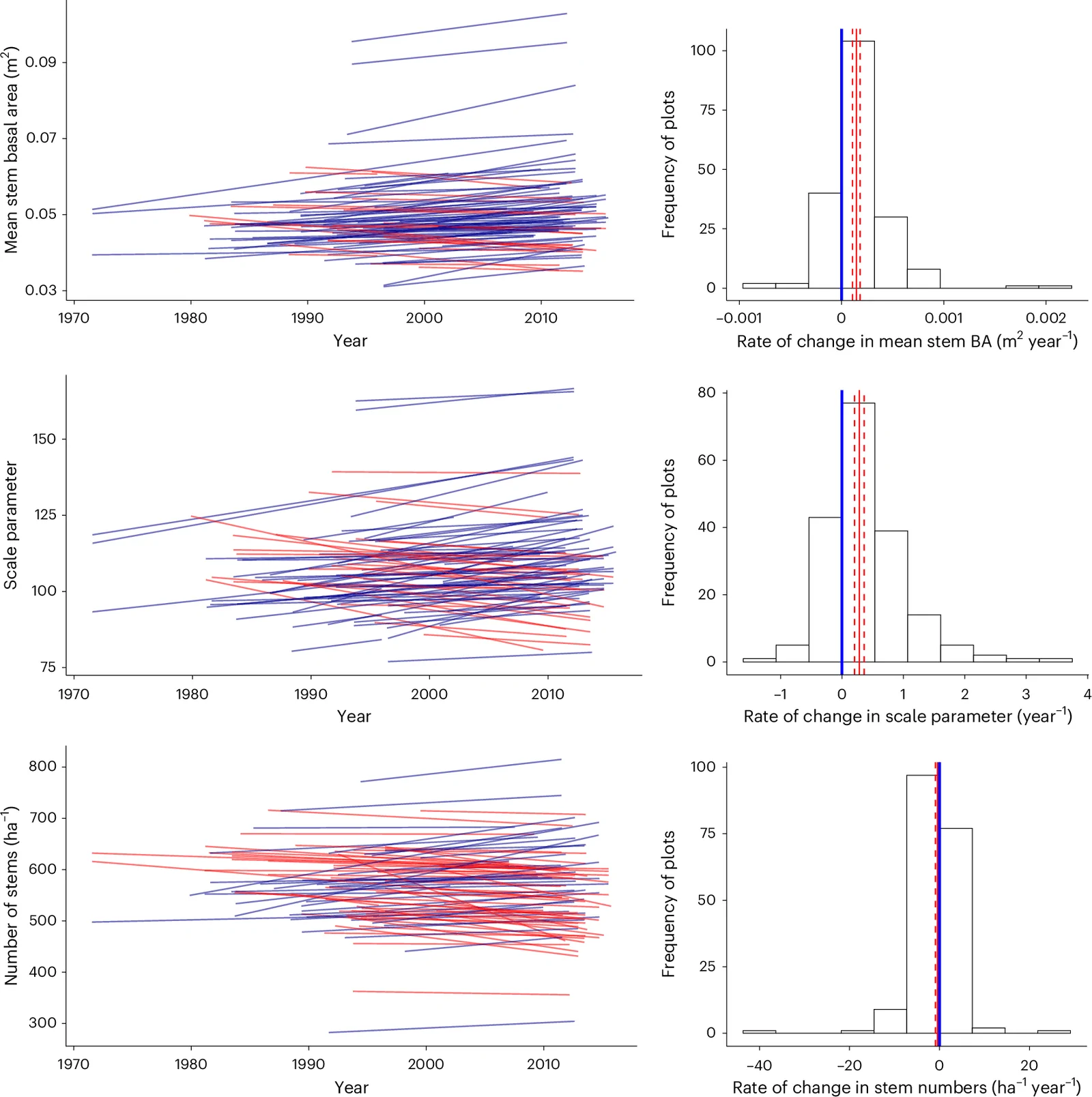
Changes in mean stem BA, scale parameter and stem numbers across mature Amazonian forests. Left: individual plot-level linear trends in size structure parameters across the full interval each plot was censused for. For visualization purposes, only 92 of the 188 plots are included, with the most strongly weighted plots based on area and monitoring period length included. Positive trend lines are coloured blue, and negative trend lines are coloured red. Right: annual rate of change of size structure parameters. Red vertical lines show the overall bootstrapped mean (solid lines) and 95% CI (dashed lines). Blue lines are positioned at 0, that is, no change.

**Extended Data Fig. 1:**
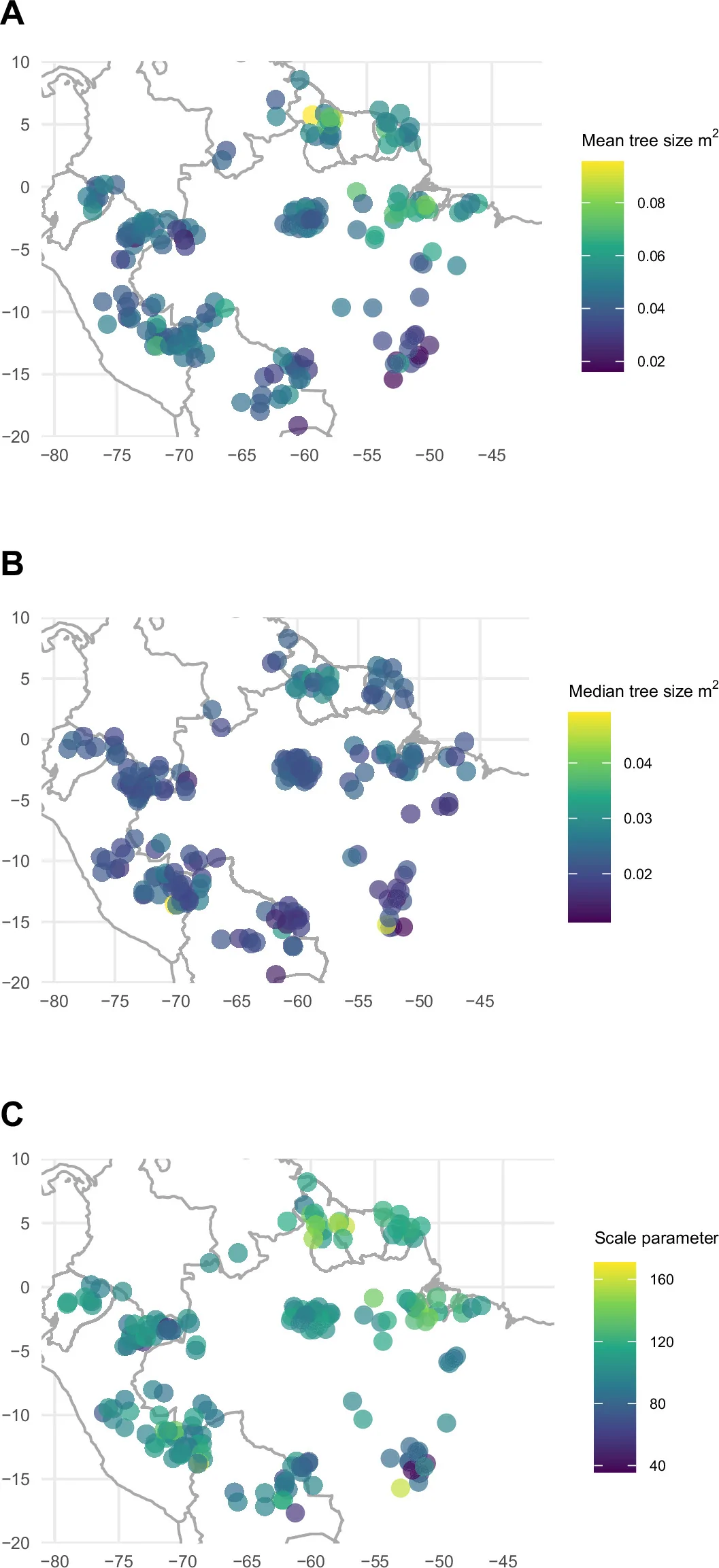
Spatial distribution and trends of forest structure across Amazonian forests. Distribution of mean (a) and median tree size (b), measured in basal area terms, and scale parameter (**c**) calculated per inventory plot across Amazonia.

**Extended Data Fig. 2:**
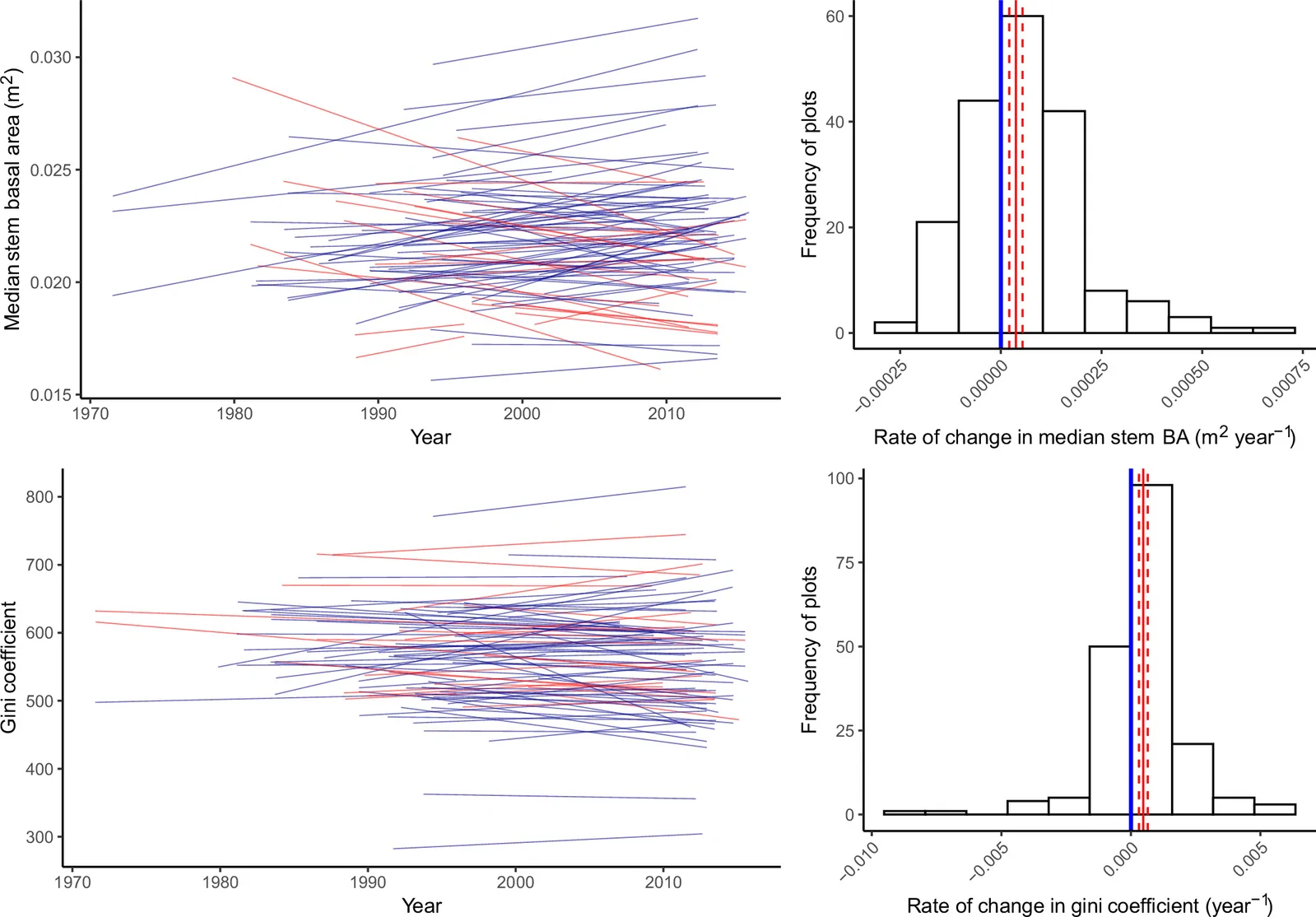
Changes in median stem BA and gini coefficient across mature Amazonian forests. Left: individual plot-level linear trends in size structure parameters across the full interval each plot was censused for. For visualisation purposes, only 92 of the 188 plots are included, with the most strongly weighted plots based on area and monitoring period length included. Positive trends lines are coloured blue, and negative trend lines are colored red. Right: Annual rate of change of size structure parameters. Red vertical lines show the overall bootstrapped mean (solid lines) and 95% CI (dashed lines). Blue lines are positioned at zero, that is no change.

At the stand level, mean tree size has increased across the whole domain of Amazon forests (Figs. 2a, 3 and 4). Mean tree BA increased at a rate of 1.45 × 10^−4^ m^2^ yr^−1^, a 3.3% gain per decade compared to initial sizes of average 4.78 × 10^−2^ m^2^. Median tree BA increased by 1.9% per decade, while maximum tree size increased by 5.8% per decade (Table 1 and Extended Data Figs. 1 and 2).

**Table 1:** Trends in tree size across the Amazon basin

	Mean (*t*_0_)	Absolute annual trend	Relative annual trend (%)	
Stem BA				
Mean (m^2^)	4.78 × 10^−2^	1.45 × 10^−4^ (1.08 × 10^−4^ to 1.82 × 10^−4^)	0.33 (0.24 to 0.41)	↑
Median (m^2^)	2.18 × 10^−2^	3.75 × 10^−5^ (2.11 × 10^−5^ to 5.38 × 10^−5^)	0.19 (0.12 to 0.27)	↑
Maximum (m^2^)	1.03	4.55 × 10^−3^ (1.92 × 10^−3^ to 7.23 × 10^−3^)	0.58 (0.38 to 0.78)	↑
Total BA (m^2^ ha^−1^)	26.53	5.52 × 10^−2^ (3.49 × 10^−2^ to 7.54 × 10^−2^)	0.24 (0.16 to 0.33)	↑
Gini coefficient	0.57	4.78 × 10^−5^ (3.09 × 10^−4^ to 6.48 × 10^−4^)	0.09 (0.06 to 0.1)	↑
Shape parameter (*γ*)	0.92	8.17 × 10^−4^ (3.46 × 10^−4^ to 1.28 × 10^−3^)	0.09 (0.04 to 0.14)	↑
Scale parameter (*β*)	104.99	0.28 (0.20 to 0.36)	0.30 (0.22 to 0.37)	↑
Stem numbers				
Total stems (ha^−1^)	565.74	*−0.45 (−0.90 to0.01)*	*−0.06 (−0.14 to0.02)*	
<200 mm (ha^−1^)	359.07	−0.54 (−0.93 to −0.15)	−0.12 (−0.22 to −0.02)	**↓**
200–399 mm (ha^−1^)	164.20	*−0.08 (−0.25 to0.09)*	*0.04 (−0.08 to0.17)*	
>400 mm (ha^−1^)	42.47	0.18 (0.11 to 0.23)	0.66 (0.40 to 0.92)	↑
Mean stem BA by size class				
<200 mm (m^2^)	1.56 × 10^−2^	1.3 × 10^−5^ (7.5 × 10^−6^ to 1.9 × 10^−6^)	0.09 (0.05 to 0.12)	↑
200–399 mm (m^2^)	6.97 × 10^−2^	*2.02* × *10*^−*5*^ *(−3.7* *×* *10*^−*6*^ *to4.4* × *10*^−*5*^*)*	*0.04 (−0.01 to0.08)*	
≥ 400 mm (m^2^)	0.26	4.6 × 10^−4^ (2.5 × 10^−4^ to 6.6 × 10^−4^)	0.19 (0.11 to 0.27)	↑
Total stem BA by size class				
< 200 mm (m^2^ ha^−1^)	5.60	*−4.16* × *10*^−*3*^ *(−1.02*× *10*^−*2*^ *to1.82* × *10*^−*3*^*)*	*−0.03 (−0.14 to0.07)*	
200–399 mm (m^2^ ha^−1^)	9.83	*−2.39* × *10*^−*3*^ *(−1.26* × *10*^−*2*^ *to7.92* × *10*^−*3*^*)*	*0.07 (−0.05 to0.20)*	
≥400 mm (m^2^ ha^−1^)	11.10	6.23 × 10^−2^ (4.61 × 10^−2^ to 7.84 × 10^−2^)	0.84 (0.60 to 1.09)	↑

Bootstrapped mean and 95% CI (in brackets) of absolute and relative trends in tree size parameters. Non-significant trends are in italics. Arrows show direction of trends for those that are significant.

Larger trees gained more in absolute, but not relative, terms. The rate of change in mean tree size was three times greater than the increase in the median tree size (Table 1). This resulted in a greater inequality in the area occupied by each tree shown by a significant increase of 1% per decade in the Gini coefficient (Table 1 and Extended Data Fig. 2).

**Extended Data Fig. 3:**
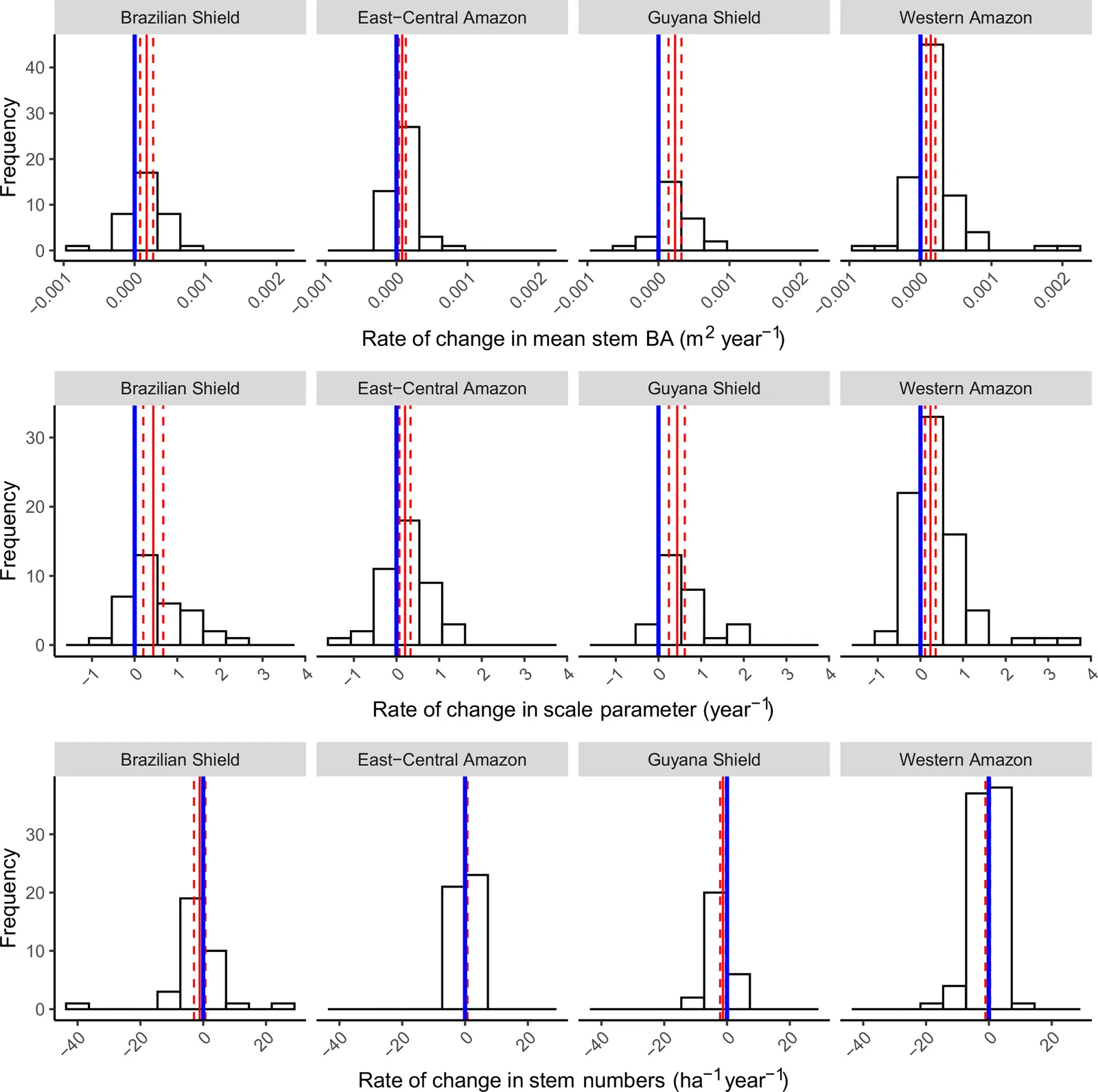
Changes in forest structure for the four Amazonian biogeographic regions. Histograms of change in mean stem BA, the scale parameter, and stem numbers per ha by biogeographic region, showing consistent directional change in size distribution parameters across all four regions. Red vertical lines show the overall bootstrapped mean (solid lines) and 95% CI (dashed lines). Blue lines are positioned at zero, that is no change.

The scale parameter of the Weibull distribution increased by 3% per decade (Figs. 2b, 3 and 4) suggesting an increase in the proportion of large stems relative to small stems and an increase in the spread of stem diameters across the distribution. This trend was widespread across the Amazon basin (Fig. 2b) and observed across all four biogeographic regions (Extended Data Fig. 3 and Extended Data Table 1). We further observed a significant increase of 1% per decade in the shape parameter suggesting a decline in the frequencies of the smallest stems and a shift towards a less right-skewed distribution (Table 1).

We found no evidence of change in total stem numbers (Fig. 4 and Table 1), but the numbers of small stems (*D* < 200 mm, *D* = diameter) and understorey stems have both declined at rates of 1.2% and 3.6% per decade, respectively (Table 1 and Extended Data Table 2). In parallel, the number of large stems (*D* > 400 mm) has increased at a rate of 6.6% per decade (Table 1). This is consistent with the observed changes in the Weibull distribution parameters. The number of medium-sized stems (*D* = 200–299 mm) has not changed.

**Fig. 5:**
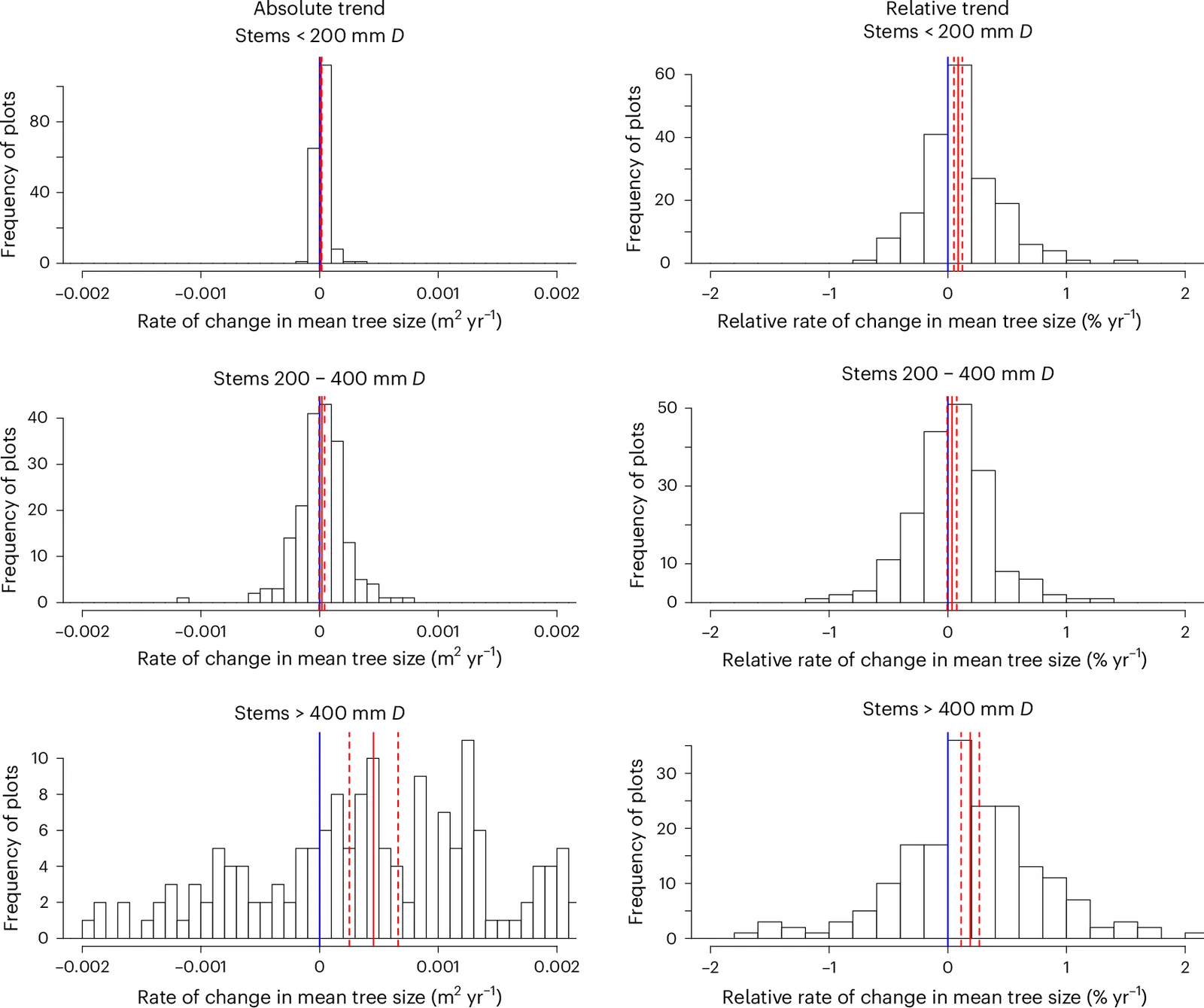
Histograms of linear slopes of absolute and relative change in tree size in Amazon plots as a function of time within tree size classes. Comparison between trends in tree size within different size classes (*D* < 200 mm, *D* = 200–399 mm and *D* ≥ 400 mm). Red solid line and dashed lines represent bootstrapped mean and 95% CI, zero is shown by the red line. To help visualize trends in absolute terms, plots that show annual rates of change < −0.002 m^2^ yr^−1^ or > 0.002 m^2^ yr^−1^ are omitted from the graph (59 plots, *D* ≥ 400 mm; 1 plot, *D* = 200–399 mm). Note that although the increase in tree size is more evident within large trees (*D* ≥ 400 mm) in absolute terms, the trends in size are similar in relative terms regardless of the size class.

**Extended Data Fig. 4:**
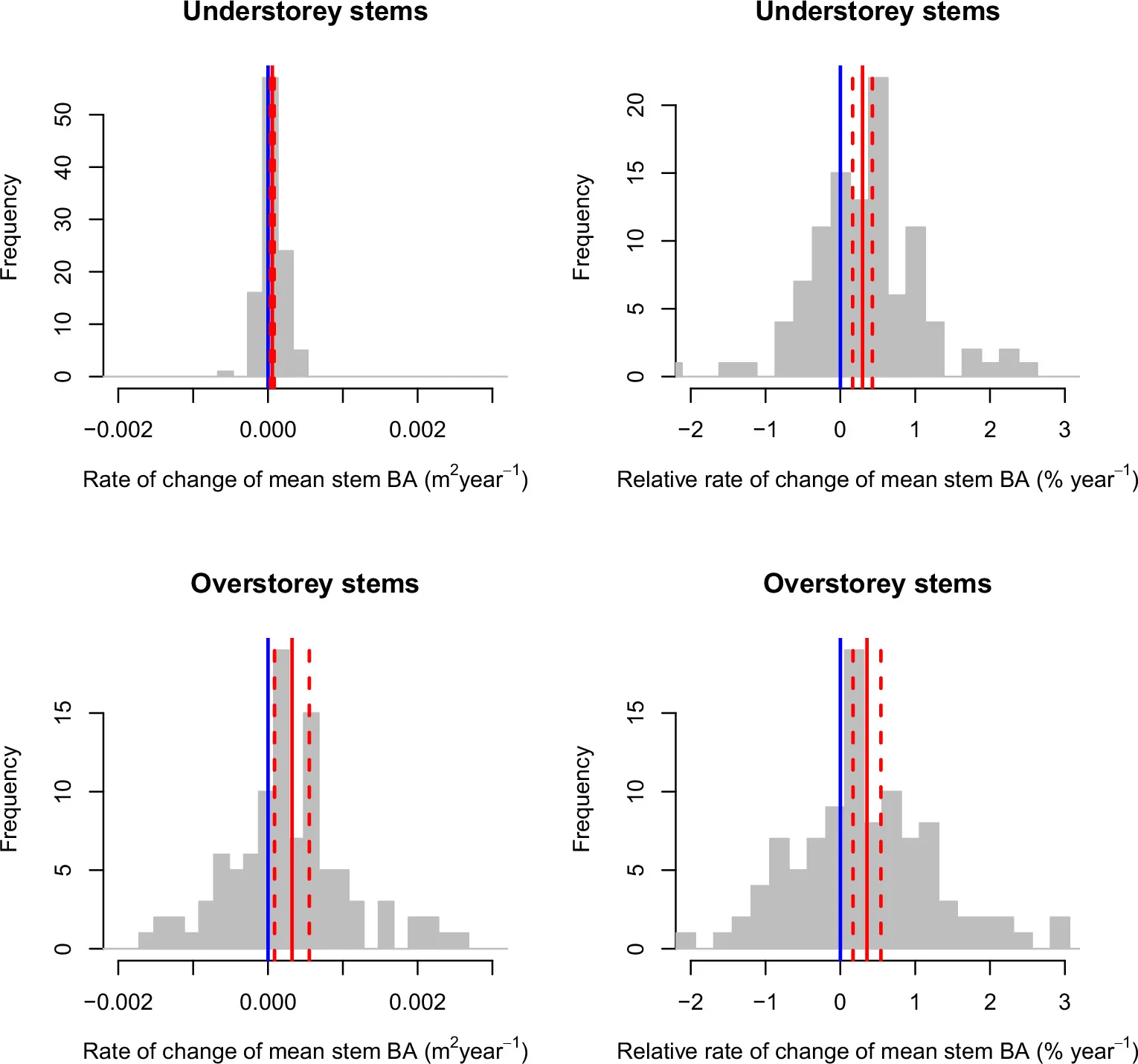
Histograms of linear slopes of absolute and relative change in mean stem BA in Amazon plots between different canopy strata. Red solid line and dashed lines represent bootstrapped mean and 95% CI, while the blue line shows 0. Canopy stratum was defined using the Ideal Tree Distribution (ITD) model (5). Note that trends in tree size for overstorey and understorey differ in absolute but not in relative terms.

The increase in tree size was observed across the whole community. Mean tree BA increased for both the smallest (*D* < 200 mm) and largest (*D* ≥ 400 mm) size classes (Table 1), as well as for understorey and overstorey trees (Extended Data Fig. 4 and Extended Data Table 2). In absolute terms, increases in BA are greater for larger size classes and canopy trees. However, in relative terms, the increase in size is not notably different when comparing large and small trees (Table 1 and Fig. 5).

**Extended Data Fig. 5:**
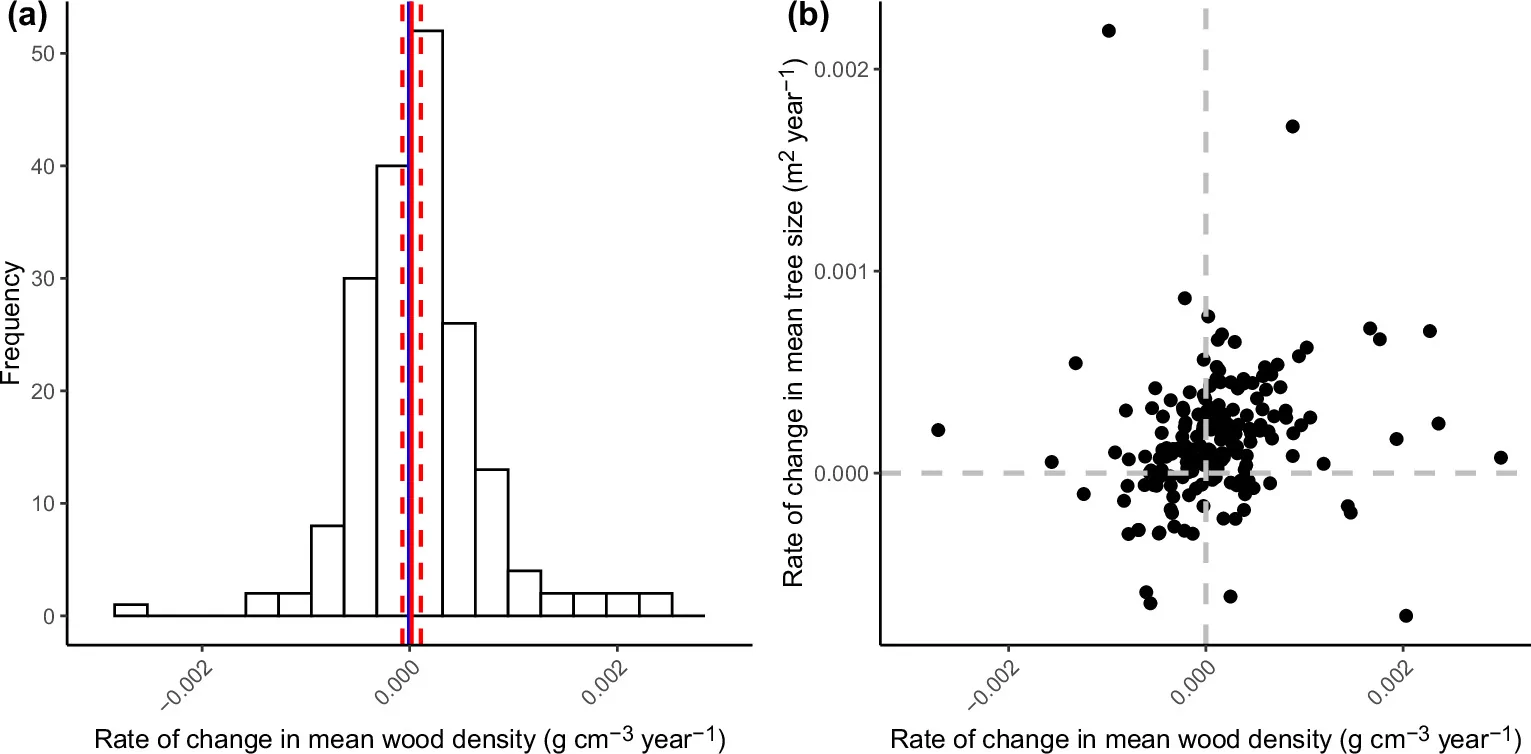
Trends in plot-level wood density. (**a**) Histogram showing the distribution of rates of change in plot-level mean wood density. Overall trends in wood density are not significantly different from zero (mean = 1.51 ×10^−5^, 95% CI = -7.28 x 10^−5^; 1.01 x 10^−4^). (**b**) Relationship between trend in mean tree size and trend in mean wood density (two-sided t-test, t = 7.32, p-value = 0.16, R^2^ = 0.005). If forests were under late successional recovery, an increase in mean tree size would be associated with an increase in wood density.

Smaller stems increased in mean stem BA, with a positive trend for relative tree size, but no change was observed in their total BA, likely due to a reduction in the frequency of smaller stems (Table 1). By contrast, the larger stems increased in size and in frequency. Thus, total BA also increased by 8.4% per decade for stems in the largest size class and by 2.8% per decade for overstorey stems (Table 1 and Extended Data Table 2). We found no evidence of a directional trend in plot-level wood density (Extended Data Fig. 5).

## Discussion

Our results show clear and pervasive changes in the structure of Amazonian forests over the recent decades. We find that tree size has been increasing across all size classes in the tree community and across different canopy strata, although in absolute terms this change has been greatest for the largest stems. As a result, we observe directional changes in the overall size distributions of Amazonian forests, with distributions becoming increasingly left-skewed, reflecting an increasing dominance and abundance of large stems. Concurrently, stem frequencies in the smallest size classes have declined. Overall, our findings suggest that the consistent increases in BA—and, by extension, biomass—across Amazonian forests is increasingly concentrated in the largest trees.

The observed increases in tree size and BA are consistent with previous studies reporting a carbon sink across tropical forests stimulated particularly by CO_2_ fertilization^1^. Given the increases in tree size observed across the community, our findings offer support for a combination of a resource-driven boost for canopy trees (winners take all), and a reduction in growth suppression among understorey trees (carbon-limited benefit). It is worth noting that we find no evidence for declines in tree size or BA, suggesting that any negative climate-driven impacts on larger trees have so far been outweighed by the effects of increase in resources.

**Extended Data Fig. 6:**
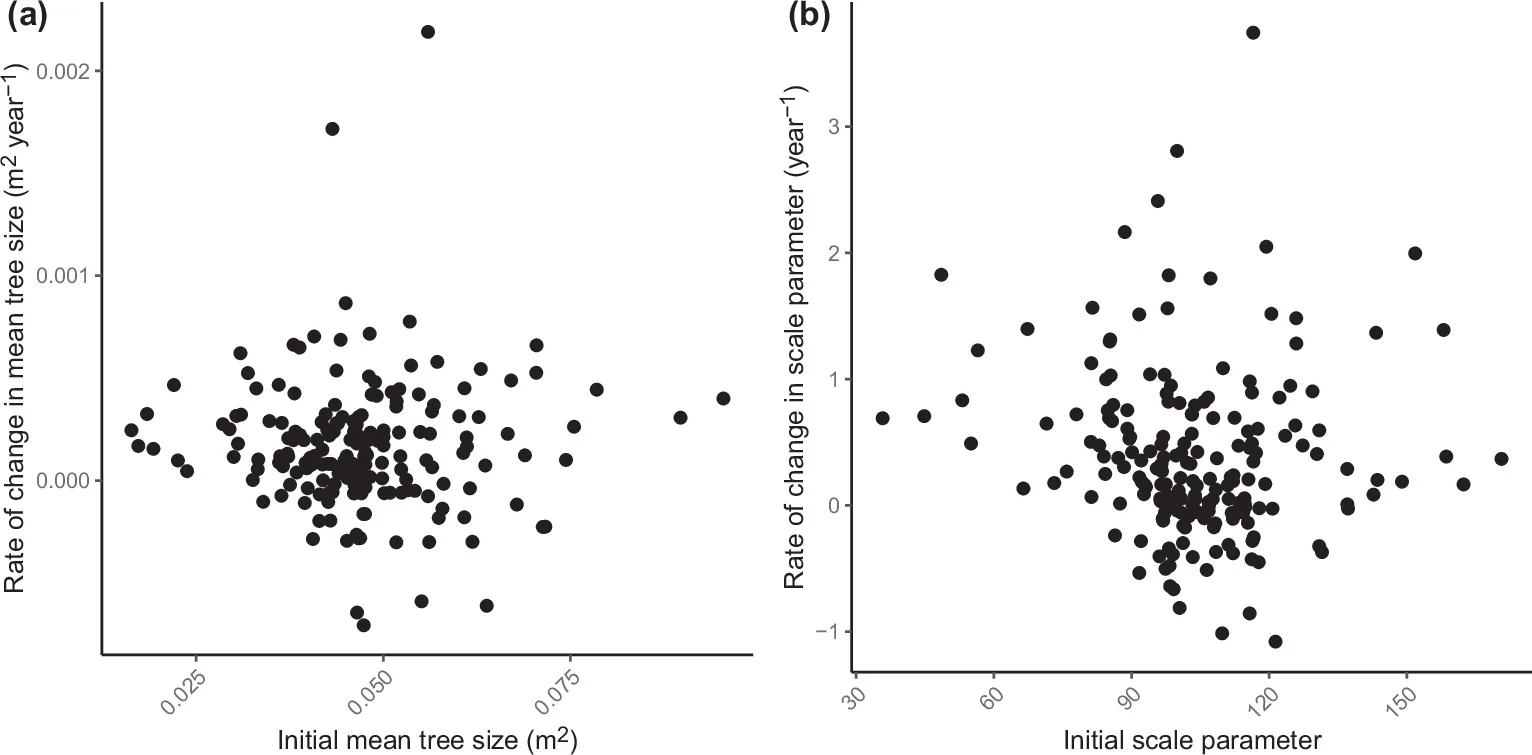
Effect of initial values of structural parameters on their rates of change. (**a**) Relationship between initial mean tree size and rate of change in mean tree size (two-sided t-test, t = 1.8, p-value = 0.89, R^2^ = 0) (**b**) Relationship between initial scale parameter and rate of change in scale parameter (two-sided t-test, t = 2.7, p-value = 0.21, R^2^ = 0.003). If forests were under successional recovery, we would expect the increase in size to be more pronounced in forests with smaller initial mean tree size and lower scale parameter values.

Our results are not consistent with trends that would be expected if late successional recovery from past disturbance—due to occupation of these forests by early Amazonian peoples^31–33^ or natural disturbances^34,35^—was the dominant driver of change. As succession advances, self-thinning takes place and the number of trees drops as space is occupied by fewer larger trees, leading to an increase in mean tree size^36^. Simultaneously, there is floristic turnover from lighter-wooded pioneers to denser-wooded late successional species^36^. If our results were primarily driven by recovery from disturbance, we would expect the increase in size to be more pronounced in forests at earlier stages of succession with smaller initial mean tree size, and to observe floristic compositional shifts towards lighter wooded species. However, we find no relationship between the change in tree size and floristic turnover towards denser-wooded trees (Extended Data Fig. 5), consistent with a previous analysis of compositional change across mature Amazonian forests^37^, and the increase in tree size was independent of the initial mean tree size (Extended Data fig. 6). Overall, our analyses indicate that the pervasive increase in tree size observed here is unlikely to be driven by an Amazon-wide recovery from previous disturbances.

The observed patterns match the expectations from increase in resources either by CO_2_ fertilization or by nitrogen deposition. Although atmospheric nitrogen deposition is a major driver of change in forests of temperate regions^38^, there is weaker evidence of its impact on tropical mature forests, particularly in remote regions^39^. First, mature Amazonian forests tend to be phosphorus and not nitrogen limited^40^, meaning that increases in nitrogen would not necessarily translate into greater productivity^39,41^. Second, although nitrogen deposition rates are expected to increase, they remain quite low^7,42^ and concentrated across the fragmented southern border of Amazonia^43^. On the contrary, atmospheric CO_2_ has progressively increased year after year globally and across all tropical forests, consistent with the Amazonian-wide tree size increase^9^ (Fig. 2). Thus, we conclude that the increase in atmospheric CO_2_ is the most likely, although potentially not only, driver of the observed increase in tree size.

The winners-take-all hypothesis predicts that under greater resource availability, the asymmetric competition for light intensifies and the competitive advantage for large canopy trees increases^11^. Consistent with this hypothesis, we observe an increase in the dominance of large canopy trees across multiple metrics, including maximum stem size and total BA. This indicates that asymmetric growth responses are causing biomass to become increasingly concentrated in the largest stems. However, an increase in asymmetric competition alone is insufficient to explain all the observed trends. Although large trees increased the most in absolute terms, relative changes in size were approximately equal across size classes and strata (Fig. 5, Table 1 and Extended Data Fig. 4). Size increases in smaller and understorey trees are consistent with experimental studies in which additional atmospheric CO_2_ alleviated suppression of understorey trees, including releasing them from negative carbon balance at low light levels^26,44,45^. We demonstrate this for forest trees in a non-experimental setting, and our findings add to evidence suggesting an important role for smaller and understorey trees as a long-term component of the forest carbon sink^46,47^.

The increasing dominance of large trees is consistent with the evidence of a substantial carbon sink in many forests^2,3^ and runs counter to the large-trees-lose hypothesis that larger trees should be decreasing in abundance because of their greater susceptibility to climate-related drivers of mortality, such as drought and windthrow^29,30,48^. Across Amazonia, we find no evidence for declines in the abundance or size of the largest trees. This suggests that, while mortality risks for large trees may be increasing^29^, the impact of this on forest structure has been outweighed by forest responses to increased CO_2_.

While the increase in tree size was observed for different size classes, we find diverging trends in stem frequencies among size classes. The per-area density of large stems has increased, but the numbers of smaller stems have declined. This may be related to the stabilization of recruitment rates across Amazonia^3^, meaning that increases in growth rates are not being matched by ingrowth from recruitment. However, declines in stem numbers may also be linked to rising mortality rates, with increasing in growth rates expected to accelerate tree life cycles leading to rise in mortality rates^49,50^. Similar changes in stand structure have been observed in temperate forests and are thought to be related to changes in competitive self-thinning relationships^51^. Regardless of the cause, declines in stem frequencies at smaller sizes have implications for the permanence of the observed trends and for the resilience of the overall ecosystem.

Our findings offer an important benchmark for understanding historic and future dynamics of the Amazonian carbon sink. Over recent decades, both growth rates and mortality rates have increased in Amazonian forests, with the increases in mortality lagging the increases in growth^3^. Our results are consistent with these changes. However, the increases in tree size may diminish and cease in coming decades, consistent with recent projections indicating future declines in the tropical forest carbon sink, if carbon losses increase^2^. Our findings provide a reference point for developing projections further, for example, by revealing that biomass is increasingly concentrated in the largest trees. The future growth and mortality dynamics of large trees will therefore be increasingly critical for the trajectory of the net carbon balance^52^.

We show that the structure of Amazonian forests is changing, with important consequences for the functioning and resilience of this system. Our results can be understood as a sign of the resilience of Amazonian forests, showing that any impacts of climate change on larger trees have been more than alleviated by the effects of CO_2_ fertilization. Whether these benefits are sufficient to counteract expected future increases in climate-related risks for the largest trees—which are more susceptible to heat, drought, lightning and windthrow—remains to be seen.

## Methods

### Vegetation data set

To describe temporal trends of forest stand structure across lowland (<1,000 m above sea level) tropical South American moist terra firme forests, we selected all long-term permanent tree monitoring plots meeting these criteria from the Amazon Forest Inventory Network (RAINFOR), an international collaboration conducting long-term monitoring of forest inventory plots^53^. Plot data were accessed via the ForestPlots.net repository^54,55^. These 188 plots had an average size of 1.2 ha (ranging 0.4 to 12 ha). Plots were monitored on average for 13 years (ranging 2 to 30 years), and the mean census interval length was 2.98 years. The monitoring period varied between 1971 and 2015 among plots; the mean date of the first and last census was 1996 and 2010, respectively. The plots used in this study have no sign of substantial human disturbance and show no detectable legacy effect of past fire disturbance on tree composition^56^. Plots with known anthropogenic disturbances such as selective logging and fire were excluded from the analyses. All trees ≥10 cm diameter were marked, had their diameter (*D*) measured at 1.3 m from the ground when no trunk deformities are present, and mapped following a standardized protocol^57^. Lianas and coarse herbs (*Phenakospermum*) were excluded from the analyses. Palms were included in the analysis. As palms do not have radial growth, variation in the abundance of palms within plots may influence the magnitude of trends in structural parameters; we tested for this potential influence in Appendix 1 in the Supplementary Information. Further methodological details have been published elsewhere^3^.

One complexity when monitoring tropical trees is that they may have buttresses or deformities that can extend above the standard point of measurement (POM, 1.3 m) during the monitoring period. When there is any deformity compromising the cylindrical shape of the trunk at 1.3 m, such as buttresses, the POM is placed at higher parts of the trunk, above any deformities where the trunk is cylindrical^57,58^. Over time if buttresses or deformities occasionally further develop, the POM must be raised so that diameter measurements are not erroneously inflated. Such changes lead to discontinuities in growth data for individual trees. To deal with this, we use a sequence of the mean *D* estimated between the first and last POMs across the monitoring period, a solution reported in several previous studies^3,59,60^. We tested for any effect of the non-continuity of some POMs, and of the procedure to correct for it, on our results (Appendix 2 in the Supplementary Information).

### Size structure

To investigate trends in tree size across Amazon basin forests, we characterize the size structure of each census by (1) the mean, median and maximum stem BA; (2) total stand BA per hectare; (3) the Gini coefficient of inequality; (4) the stem diameter frequency distribution described by the shape (*γ*) and scale (*β*) parameters of a two-parameter Weibull distribution; (5) the mean stem BA in each of three size classes, *D* < 200 mm, *D* = 200–399 mm and *D* ≥ 400 mm; (6) the mean stem BA of overstorey and understorey trees (see Appendix 3 in the Supplementary Information for methods to classify canopy status); (7) the mean number of stems per hectare; (8) the mean number of stems per hectare in each of three size classes, *D* < 200 mm, *D* = 200–399 mm and *D* ≥ 400 mm; and (9) mean stand-level wood density.

The Gini coefficient, used to quantify inequality, is derived from the Lorenz curve^61^, which in forest ecology is used to describe the distribution of the total area of a plot occupied by trees^62^. It represents the area between a hypothetical line where all individuals occupy the same area in a plot and the Lorenz curve, which is the cumulative proportion of area occupied by each tree as a function of the cumulative proportion of the number of trees. Thereby, if all individuals occupy the same area, Gini is equal to 0, while a completely unequal situation will be represented by Gini = 1 (ref. ^62^). We calculated the Gini coefficient in each census using the ineq R package version 0.2-13 (ref. ^63^).

A two-parameter Weibull distribution was fitted to stem *D* frequency distributions, with the equation

1 $$f\left(x\right)=\frac{\gamma }{\beta }{\left(\frac{x}{\beta }\right)}^{(\gamma -1)}\exp -{\left(\frac{x}{\beta }\right)}^{\gamma }\gamma ,\beta > 0$$

where *γ* is the shape parameter and *β* is the scale parameter. The Weibull distribution is well suited to describing stem *D* frequency distributions, as it fits a wide range of distribution shapes^19,64^. The scale parameter *β* controls the spread of the distribution; higher scale parameter values indicate stem *D* distributions with a larger spread of stem *D* values and a higher proportion of large stems relative to small stems. The shape parameter *γ* controls the shape of the distribution. Shape parameter values <1 result in a right-skewed ‘reverse-J’ distribution with steadily decreasing stem frequencies. As shape parameter values increase, the distribution becomes less right-skewed, and the distribution approximates a normal distribution where shape parameter values are ~3.

If large trees are gaining as predicted by the winners-take-all hypothesis, we expect the scale parameter *β* to increase, with stem *D* distributions showing an increase in the spread of stem sizes and a higher proportion of larger stems relative to small stems. We would also expect an increase in the shape parameter *γ* indicating a decline in the frequency of smaller stem sizes and a shift towards a less right-skewed distribution.

To assess whether any observed trends are primarily driven by recovery from disturbance, we also analyse trends in mean stand-level wood density. As forests recover from disturbance, there is floristic turnover from lighter-wooded pioneers to denser-wooded late successional species^36^, so if Amazonian forests are recovering from disturbance, we would expect to observe a change in floristic composition towards heavier wooded species. Wood density data were extracted from the Global Wood Density database^65^^,66^. Wood density values were obtained at species level, where possible, and otherwise at the level of genus or family. Stems which could not be assigned a wood density value at these levels were removed from this particular analysis (2.9% of total stems).

### Analytical approach

We investigated mean linear trends of each of the above stand structure parameters across the whole dataset. First, the linear trends for the individual plots were calculated as the linear slope of an ordinary least-squares regression of the size–structure parameters as a function of time (the date when the census took place). Then, to test whether the overall response across the Amazon basin differed from 0, bootstrapped mean and 95% CI were obtained by randomly resampling values of plot-level trends, with replacement, across all plots 10,000 times^67,68^. For the mean, median and maximum tree BA, total BA, Gini coefficient, shape parameter (*γ*), scale parameter (*β*) and stem density (stems per hectare), we also analysed trends by biogeographic region (Appendix 4 in the Supplementary Information). These analyses were repeated in relative terms, where size parameters where relativized by the size parameter in the first census. We weighted plots by the square root of plot area times the monitoring period to reduce the influence of potential stochastic changes, which are most likely to affect small plots and plots monitored over short monitoring periods^3,60^.

### Reporting summary

Further information on research design is available in the Nature Portfolio Reporting Summary linked to this article.

## Supplementary information

Supplementary Information Supplementary Appendices 1–4.

Reporting Summary

## Data Availability

The source data underlying the analyses are available at https://doi.org/10.24433/CO.0443999.v2.

## Extended data

**Extended Data Table 1:** Regional trends in tree size parameters

	Biogeographic region	Mean (t_0_)	Relative annual trend (%)
Mean stem BA (m^2^)	Brazilian Shield	0.04	0.48 (0.23 | 0.72)
	Western Amazon	0.05	0.35 (0.20 | 0.50)
	East Central Amazon	0.05	0.18 (0.08 | 0.28)
	Guiana Shield	0.06	0.40 (0.24 | 0.55)
Median stem BA (m^2^)	Brazilian Shield	0.02	0.36 (0.08 | 0.64)
	Western Amazon	0.02	0.18 (0.06 | 0.29)
	East Central Amazon	0.02	*0.11 (-0.02 | 0.24)*
	Guiana Shield	0.02	0.24 (0.03 | 0.44)
Maximum stem BA (m^2^)	Brazilian Shield	0.92	0.45 (0.00 | 0.94)
	Western Amazon	1.05	0.70 (0.34 | 1.05)
	East Central Amazon	1.11	0.52 (0.24 | 0.79)
	Guiana Shield	0.92	0.49 (0.13 | 0.84)
Total BA (m^2^ ha^−1^)	Brazilian Shield	21.78	*0.33 (-0.04 | 0.70)*
	Western Amazon	25.97	0.25 (0.12 | 0.38)
	East Central Amazon	28.30	0.23 (0.12 | 0.35)
	Guiana Shield	29.35	0.16 (0.01 | 0.32)
Gini Coefficient	Brazilian Shield	0.54	0.12 (0.01 | 0.23)
	Western Amazon	0.56	0.13 (0.08 | 0.18)
	East Central Amazon	0.58	0.03 (0.00 | 0.07)
	Guiana Shield	0.59	*0.05 (-0.01 | 0.13)*
Shape parameter (γ)	Brazilian Shield	0.92	*0.19 (-0.01 | 0.40)*
	Western Amazon	0.93	*0.05 (-0.02 | 0.12)*
	East Central Amazon	0.91	0.08 (0.00 | 0.16)
	Guiana Shield	0.90	0.14 (0.00 | 0.27
Scale parameter (β)	Brazilian Shield	90.76	0.55 (0.27 | 0.82)
	Western Amazon	102.26	0.27 (0.14 | 0.40)
	East Central Amazon	108.50	0.18 (0.06 | 0.30)
	Guiana Shield	119.85	0.37 (0.20 | 0.52)
Number of stems (ha^−1^)	Brazilian Shield	539.78	*-0.12 (-0.44 | 0.20)*
	Western Amazon	578.94	*-0.07 (-0.20 | 0.07)*
	East Central Amazon	577.56	*0.06 (-0.03 | 0.15)*
	Guiana Shield	528.78	-0.22 (-0.38 | -0.05)

Bootstrapped mean and 95% CI (in brackets) of relative annual trends in tree size parameters by biogeographic region. Non-significant trends are in italics.

**Extended Data Table 2:** Trends in tree size across the Amazon basin by tree stratum

	Mean (t_0_)	Absolute trend	Relative trend (%)
**Mean stem BA by stratum**			
Understorey (m^2^)	2.00 x 10^−2^	5.74 x 10^−5^ (-3.18 x 10^−6^ | 8.27 x 10^−5^)	0.30 (0.16 | 0.43)
Overstorey (m^2^)	8.67 x 10^−2^	3.21 x 10^−4^ (8.47 x 10^−5^ | 5.56 x 10^−4^)	0.35 (0.17 | 0.54)
**Total stem BA by stratum**			
Understorey (m^2^ ha^−1^)	6.86	*-0.01 (-0.03 | 0.01)*	*-0.05 (-0.62 | 0.51)*
Overstorey (m^2^ ha^−1^)	19.79	0.05 (0.03 | 0.07)	0.28 (0.15 | 0.40)

Bootstrapped mean and 95% CI (in brackets) of absolute and relative trends in tree size parameters. Non-significant trends are in italics.

## Code availability

The codes underlying the analyses are available at https://doi.org/10.24433/CO.0443999.v2.
