# Supplementary material for: Increasing tree size across Amazonia
Source: Nat Plants. 2025 Sep 25;11(10):2016–25. doi: 10.1038/s41477-025-02097-4 (PMC12537487; doi:10.1038/s41477-025-02097-4)
Supplement: Supplementary file 1 — Supplementary Appendices 1–4. [file 41477_2025_2097_MOESM1_ESM.pdf]

nature plants

Supplementary information https://doi.org/10.1038/s41477-025-02097-4

Increasing tree size across Amazonia

In the format provided by the authors and unedited

Increasing tree size across Amazonia

Supplementary information

Appendix 1 – The influence of palm abundance on trends in structural parameters

Palms are fundamental components of the ecology of Amazonia and are included in our data. However, it is important to note that palms do not have radial growth, thus are not expected to increase in diameter over time. Therefore, the inclusion of palms in our analysis may influence the magnitude of observed trends in structural parameters.

The percentage of palm stems in each plot ranges between 0-31%, though most plots have low palm abundance: 78% of plots have <10% of palm stems and 22% of plots have no palms.

To test for any influence of palm abundance on trends, we repeated the main analyses excluding all plots where palms represent over 10% of stems (subset used for the analyses = 146 plots) and found no change in the directionality or significance of observed trends. However, as by excluding palms we exclude stems that are unlikely to increase in size, the magnitude of some of the observed trends marginally increased. Specifically, we observed an increase in the relative trend for mean BA from 0.32 to 0.38% y^{-1}, and an increase in the relative trend for the scale parameter from 0.30 to 0.37% y^{-1}.

We further tested for any relationships between palm abundance, expressed as proportion of palms within the plot, and the plot-level trend in structural parameters and found only weak associations (maximum R^2 0.05) between palm abundance and the magnitude of plot-level trends (Table S1).

Table S1. Effect of palm abundance of trends in forest structure. Model statistics summarising the relationships between the rate of change in five structural parameters and the proportion of palms (%) within the plot. P values <0.05 are marked *.

Model (~ proportion of palms)	R-squared	p value
Mean stem BA	0.019	0.053
Stem density	0.008	0.222
Total BA	0.0003	0.813
Gini	0.0009	0.669
Scale	0.05	0.002*

Appendix 2 - The influence of measurement technique on basal area estimations

The method used to assess trees' diameter (D) across time can potentially influence results in terms of tree size change. Conventionally, tree growth is assessed based on the diameter of the tree trunk at 1.3 m above the ground (diameter at breast height - DBH), assuming the trunk is cylindrical at the point of measurement (POM). However, this is often not the case for tropical trees that commonly have buttresses or deformities extending above the 1.3 m standard POM. For those trees with irregular trunks, the POM is instead placed in higher areas of the trunk, above any deformities where the trunk is cylindrical (Sheil, 1995). Buttresses often move upwards during trees' lifespan, which implies that the POM sometimes has to be shifted towards higher parts of the trunk in advance in order to measure tree growth over long periods of monitoring (Sheil, 1995). Various approximations have been developed to estimate growth of those trees that had their POM changed (Cushman et al., 2014; Talbot et al., 2014).

In this study, for those trees that had the POM changed during the monitoring period, we use the mean between the diameter (D) where the old POM is recorded (D_{POMold}) and the D at the new POM (D_{POMnew}), so called $D_{POMmean}$ (Talbot et al., 2014). For every census the diameter sequences of D_{POMold} and D_{POMnew} are calculated based on the actual measured D and on estimations based on the ratio between D_{POMold} : D_{POMnew}. This method has the advantage of creating a continuous growth sequence for each individual tree, regardless of POM changes, and of using the actual measured taper of that tree to create such a sequence. $D_{POMmean}$ has been used in previous studies, where it was found to lead to results consistent with those from other D estimation technique (Brienen et al., 2015; Lewis et al., 2009; Talbot et al., 2014). However, because trees' circumferences tend to decrease with height as a result of taper, it is conceivable that the tendency of this method to adjust POMs down the tree stem for later censuses and adjust them up for earlier censuses may still lead to some overestimation of stand level growth rates.

Information from the tree taper is also often used to estimate D (Cushman et al., 2014). This technique consists of estimating the D at 1.3 m based on $D_{POMactual}$, the POM

height and the taper parameter, which quantifies the decay in D from 1.3 to $D_{POMactual}$ (Metcalf et al., 2009). However, taper parameter can vary considerably across species and the lack of species-specific taper parameters for tropical trees hampers a more widespread application of D estimations based on tree taper (Cushman et al., 2014).

To verify for possible bias in $D_{POMmean}$ one could use $D_{POMactual}$, i.e. the D where it was measured regardless of POM changes. However, $D_{POMactual}$ always underestimates the growth of stems with POM changes, since the D drops when POM is moved up the tree as a consequence of tree taper, creating discontinuous growth sequences (Phillips et al., 2002). POM changes are applied in advance of deformations reaching within 30 cm of the POM, thus another alternative is $D_{POMactual-1}$, i.e. estimation of D where D_{POMold} is applied for censuses up to, and including, the census when the new POM is first recorded, and D_{POMnew} is applied from the census where the POM change took place. The most highly conservative D estimation would be $D_{POMconst}$, that applies for each tree that had a POM change the mean growth rate of its size class in that plot (gr) during the first census interval calculated based on all Eudicots that did not have POM changes. For monocots and tree ferns with POM changes D_{POMnew} is applied. To estimate $D_{POMconst}$ the growth rate is calculated for each plot in one of three size classes (100-199mm, 200-399mm, $\geq$400mm D). Thus, $D_{POMconst} = D_{t1} + gr * (t_2 - t_1)$ and when $D_{t1}+gr*(t_2-t_1) > D$ of next size class, the gr of the next size class should be used.

Trends in plot mean POM over time are good indicators of whether there has been any bias in the measurement towards under or overestimating growth. An increase in mean POM height would either indicate that the field team has been conservative in estimating basal area increments and POM changes have been applied often, or that in fact there has been a real increase in the size or number of large buttressed trees which would have high POMs. Another way to assess potential biases in trend in mean tree size as a consequence of the measurement techniques is to investigate the trends in mean basal area-weighted POM height. An increase in basal area weighted POM shows that the POM has been placed higher, especially for large trees. Basal-area weighted POM dynamics are a closer indication of whether trees are being measured around the buttresses as it gives less weight to the fluctuation of small trees from census to census, and is expected to be most insightful when evaluating whether POM change protocols might be affecting stand-level basal area and biomass analyses.

Analyses

Investigating potential changes in mean POM height

We investigate potential bias in tree size estimation caused by shifts in POM. we assessed the trends in mean POM height, diameter weighted mean POM, and basal area weighted mean POM through time in terms of time elapsed (all plots with at least 2 censuses) and in terms of censuses number (plots with at least 5 censuses).

For each plot we calculated the linear slopes of mean and weighted mean POM as a function of time (years or number of censuses). The mean and the 95% CI of all plots weighted by plot sample effort (i.e. squared root of monitoring time x area for year-based analyses and squared root of plot area for census-based analyses) were estimated using non-parametric bootstrapping by randomly resampling values of plot-level mean change across all plots 10,000 times. This analysis was repeated using different ways to estimate basal area, i.e. $D_{POMactual}$, $D_{POMmean}$, $D_{POMactual-1}$ and $D_{POMconst}$.

Comparing results across different D estimation techniques

To assess whether $D_{POMmean}$ is in fact a robust D estimation to assess changes in mean tree size we also conducted two alternative analyses: (1) repeated the core analyses using $D_{POMactual}$ and (2) calculated mean tree size using a taper parameter to estimate D. we applied a general taper function:

$$d = D * e^{-b(h-1.3)} \quad \text{Eq. 1}$$

where d is the diameter at height h, D is the diameter when POM = 1.3 m and b is the taper parameter (Cushman et al., 2014; Metcalf et al., 2009). As species-specific b are not available we used a general taper parameter for all trees, which is calculated for each tree based on d and h following Cushman et al. (2014):

$$\log(b) = -2.4368 - 0.3566 * \log(d) + 0.3093 * \log(h). \quad \text{Eq. 2}$$

The taper correction was applied to all trees that had POM different than 1.3 m.

The analyses in this appendix, with exception to the repetition of the core analyses using $D_{POMactual}$, were developed using the plots with POM information available for at

least 80% of the stems: 147 plots were used to calculate D based on taper function and for the year basis analyses of POM height and 73 plots were used for census basis analyses of POM height.

Results

Investigating potential changes in mean POM height

The analyses trends in POM for different D estimation techniques show that field measurements have progressively moved higher in the trunk to avoid measurements around the buttresses. Thus, field teams have either progressively become more conservative in their interpretations of the need to avoid deformities, or trees have generally become larger and more buttressed, or both. Mean POM for $D_{POMactual}$ and $D_{POMactual-1}$ increased when giving the same weight to all trees or when calculating POM weighted by D or basal area (Table S2 and S3). The POM used for $D_{POMmean}$ and $D_{POMconst}$ tend to decrease when the same weight was applied to all individuals, or not to change when POM was weighted by tree basal area (Table S2 and S3; Figure S2).

Comparing results across different D estimation techniques

The main patterns of tree size observed using $D_{POMmean}$ (Table 1 - main text) persisted when using the more conservative $D_{POMactual}$ (Table S1). $D_{POMactual}$ underestimates growth as the POM moves up the trunk, which happens more frequently for large trees. However, we still found an increase in mean tree size within the largest size class (≥ 400 mm). The non-significant trend in tree size among overstorey trees is explained by the underestimation of tree size change among large trees when using $D_{POMactual}$.

The increase in mean tree size across the Amazon was more pronounced when calculated using D estimated from the taper function which was of 186 mm y^{-1} (95% CI 135; 239) (Figure S1), which does not differ from mean tree size calculated for the same dataset using $D_{POMmean}$ 145 mm y^{-1} (95% CI 107; 182).

Table S2 – Trends in tree size in forests across the Amazon basin using $D_{POMactual}$ to estimate basal area. Bootstrapped mean and 95% CI (in brackets) for trends in tree size for different group of trees and tree size parameters. Note that none of the estimated trends differ significantly from the trends calculated using $D_{POMmean}$ (Table 1 - main text).

	Absolute trend	N of plots
Mean	83 (47|123)	187
Median	28 (11|45)	187
**Size classes**		
< 200 mm	13 (7|19)	187
200 - 399 mm	14(-11|38)	187
≥ 400 mm	293(77|502)	187

Table S3 - Mean and 95 % CI annual trends in plot level mean POM, diameter weighted mean POM and basal area weighted POM for different diameter (D) estimation techniques across 146 Amazonian plots with at least 2 censuses and 80% of the trees with POM height recorded. $D_{POMactual}$ = actual D of the tree as measured at each point in time. $D_{actual-1}$ = D where the POM was initially recorded is applied for all censuses up to and including the census at which the new POM is first recorded, and D at new POM for all analyses from the census when the POM change took place. $D_{POMmean}$ = mean diameter value where the POM was recorded initially and the diameter at the new POM. $D_{POMconst}$ = applies the mean growth rate of the plot during the first census interval forwards. Mean trends and CI were weighted by plot sample effort (squared root of plot area x monitoring period).

	Mean (mm y^{-1})	2.5% CI	97.5% CI
POM height			
$D_{POMactual}$	6	4	7
$D_{POMactual-1}$	4	3	5
$D_{POMmean}$	-1	-2	-1
$D_{POMconst}$	-2	-2	-1
Diameter weighted POM height			
$D_{POMactual}$	3	2	4
$D_{POMactual-1}$	2	1	3
$D_{POMmean}$	-1	-1	-1
$D_{POMconst}$	-1	-1	-1
Basal area weighted POM height			
$D_{POMactual}$	10	7	12
$D_{POMactual-1}$	7	4	9
$D_{POMmean}$	-1	-2	0.2
$D_{POMconst}$	-2	-3	-1

Table S4 - Mean and 95 % CI trends per census of mean POM, diameter weighted mean POM and basal area weighted POM for different diameter (D) estimation techniques across 73 Amazonian plots. As table S2 but at a census basis for all plots with at least 5 censuses.

	Mean (mm census^{-1})	2.5% CI	97.5% CI
POM			
$D_{POMactual}$	6	2	11
$D_{POMactual-1}$	3	-2	7
$D_{POMmean}$	-3	-3	-2
$D_{POMconst}$	-3	-4	-2
Diameter weighted POM			
$D_{POMactual}$	13	6	20
$D_{POMactual-1}$	7	1	13
$D_{POMmean}$	-2	-4	-0.1
$D_{POMconst}$	-4	-6	-2
Basal area weighted POM			
$D_{POMactual}$	26	15	37
$D_{POMactual-1}$	17	7	27
$D_{POMmean}$	1	-4	6
$D_{POMconst}$	-4	-8	1

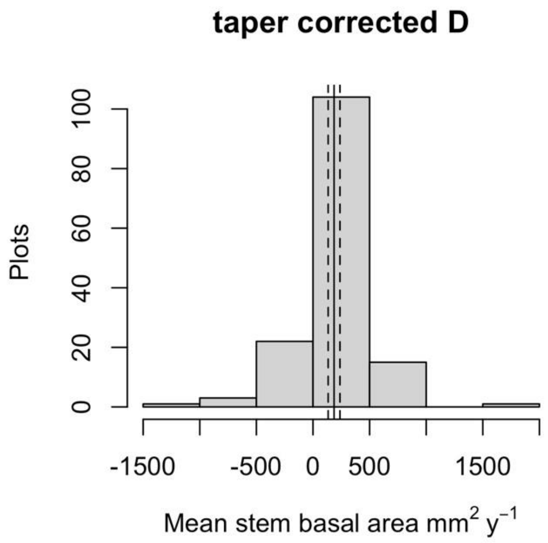

Figure S1 – Distribution of trends in mean stem basal area per plot using D estimations from the taper function.

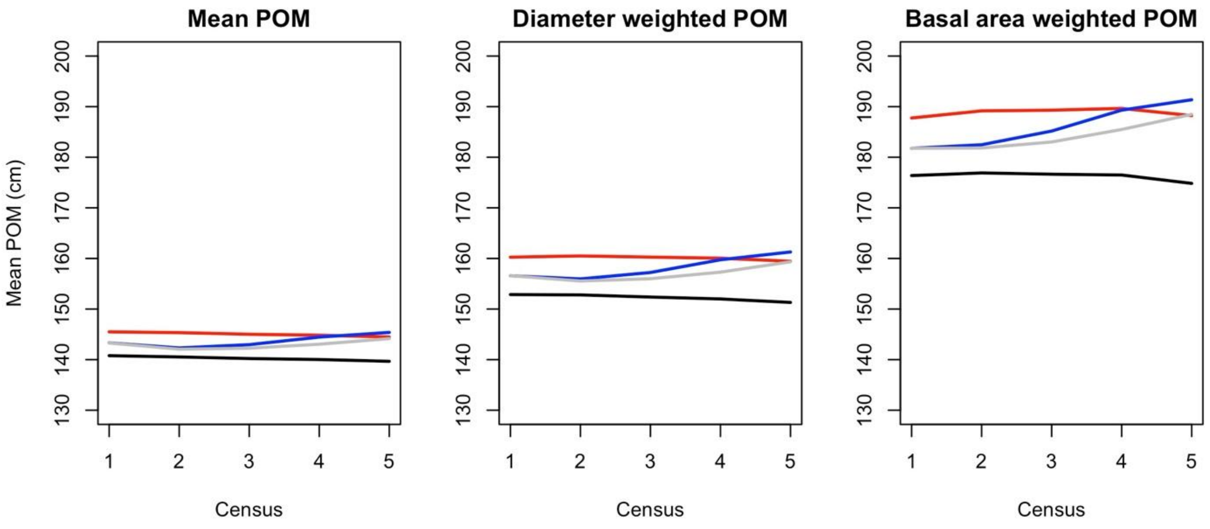

Figure S2 – Trends in mean, diameter weighed mean and basal area weighed mean POM across censuses for 73 inventory plots in the Amazon Basin. Plots had at least 5 census and 80% of trees with POM height recorded. Different colours represent POM applied to calculate different D estimation techniques: $D_{POMactual}$ (blue), $D_{POMactual-1}$ (grey), $D_{POMmean}$ (red) and $D_{POMconst}$ (black).

Appendix 3 - Canopy status

We classified each tree by their canopy status (overstorey or understorey) using the Ideal Tree Distribution (ITD) model (Purves et al., 2007). This model places the trees in the overstorey, or understorey stratum based on height and crown area. Trees are ordered from the tallest to shortest and the crown areas of the trees are summed cumulatively until this equals the ground area. These trees are classified as in the overstorey layer. All trees shorter than the smallest tree in the overstorey layer are then considered to be in the understorey. The model is based on the assumptions that: (1) the total crown area of each canopy layer is less than or equal to the ground area; (2) trees are sufficiently plastic so there should be no unused space in each canopy layer; and (3) consequentially, tree height defines which tree will be in the canopy(Bohlman & Pacala, 2012; Purves et al., 2007, 2008).

In the absence of height and crown area of each tree we estimated the height for each tree in the data in each census using regional allometric equations from Feldpausch et al. (2011). Crown area was estimated using the allometric equation of Poorter et al. (2006):

$$\text{crown area} = \exp\left[-1.853 + 1.888 \ln(\text{height})\right] \qquad \text{Eq. 2}$$

We considered the crown area of palms to be zero as that they have a different crown allometry when compared to dicotyledonous trees (Goodman et al., 2013). The model was applied at the census level, and tree height and crown area were calculated for each tree allowing canopy status could change from understorey to overstorey and vice-versa.

Canopies of tropical forests are often irregular because of gap dynamics, so to account for this patchiness we applied the model at the subplot scale(Bohlman & Pacala, 2012). Thus, the classification of trees canopy status within plots was done at the ≈ 400 m^2 subplot scale, for which subplot information was available for 106 plots plus four plots with similar subplot size (385 m^2; 450 m^2, 416 m^2 and 416 m^2). The size of the subplots used here is within the optimum plot size for predicting canopy status according to comparisons between the ITD model and ground-based and satellite data in Central America (Fauset et al., 2012; Feeley et al., 2011).

Appendix 4 – Regional-level analysis

To explore variation in trends among regions, we classify sites into four biogeographic regions defined by Feldpausch et al. (2011): (1) the Brazilian Shield; (2) the Western Amazon; (3) the East-Central Amazon; (4) the Guiana Shield. Classification of lowland Amazonian forests by biogeographic region is well-supported and reflects underlying variation in soil structure and fertility (Quesada et al., 2012) resulting in strong regional variation in forest structure and composition (Johnson et al., 2016). We analysed trends for mean, median, and maximum tree basal area, total basal area, Gini coefficient, shape parameter (γ) and scale parameter (β), stem density (stems per hectare) within each region, following the approach described in the methods.

References

Bohlman, S., & Pacala, S. (2012). A forest structure model that determines crown layers and partitions growth and mortality rates for landscape-scale applications of tropical forests. *Journal of Ecology*, *100*(2), 508–518. https://doi.org/https://doi.org/10.1111/j.1365-2745.2011.01935.x

Brienen, R. J. W., Phillips, O. L., Feldpausch, T. R., Gloor, E., Baker, T. R., Lloyd, J., Lopez-Gonzalez, G., Monteagudo-Mendoza, A., Malhi, Y., Lewis, S. L., Vásquez Martinez, R., Alexiades, M., Álvarez Dávila, E., Alvarez-Loayza, P., Andrade, A., Aragaõ, L. E. O. C., Araujo-Murakami, A., Arets, E. J. M. M., Arroyo, L., … Zagt, R. J. (2015). Long-term decline of the Amazon carbon sink. *Nature*, *519*(7543), 344–348. https://doi.org/10.1038/nature14283

Cushman, K. C., Muller-Landau, H. C., Condit, R. S., & Hubbell, S. P. (2014). Improving estimates of biomass change in buttressed trees using tree taper models. *Methods in Ecology and Evolution*, *5*(6), 573–582. https://doi.org/https://doi.org/10.1111/2041-210X.12187

Fauset, S., Baker, T. R., Lewis, S. L., Feldpausch, T. R., Affum-Baffoe, K., Foli, E. G., Hamer, K. C., & Swaine, M. D. (2012). Drought-induced shifts in the floristic and

functional composition of tropical forests in Ghana. *Ecology Letters*, *15*(10), 1120–1129. https://doi.org/10.1111/j.1461-0248.2012.01834.x

Feeley, K. J., Davies, S. J., Perez, R., Hubbell, S. P., & Foster, R. B. (2011). Directional changes in the species composition of a tropical forest. *Ecology*, *92*(4), 871–882. https://doi.org/10.1890/10-0724.1

Feldpausch, T. R., Banin, L., Phillips, O. L., Baker, T. R., Lewis, S. L., Quesada, C. A., Affum-Baffoe, K., Arets, E. J. M. M., Berry, N. J., Bird, M., Brondizio, E. S., De Camargo, P., Chave, J., Djagbletey, G., Domingues, T. F., Drescher, M., Fearnside, P. M., França, M. B., Fyllas, N. M., … Lloyd, J. (2011). Height-diameter allometry of tropical forest trees. *Biogeosciences*, *8*(5), 1081–1106. https://doi.org/10.5194/bg-8-1081-2011

Goodman, R. C., Phillips, O. L., del Castillo Torres, D., Freitas, L., Cortese, S. T., Monteagudo, A., & Baker, T. R. (2013). Amazon palm biomass and allometry. *Forest Ecology and Management*, *310*, 994–1004. https://doi.org/https://doi.org/10.1016/j.foreco.2013.09.045

Johnson, M. O., Galbraith, D., Gloor, M., De Deurwaerder, H., Guimberteau, M., Rammig, A., Thonicke, K., Verbeeck, H., von Randow, C., Monteagudo, A., Phillips, O. L., Brienen, R. J. W., Feldpausch, T. R., Lopez Gonzalez, G., Fauset, S., Quesada, C. A., Christoffersen, B., Ciais, P., Sampaio, G., … Baker, T. R. (2016). Variation in stem mortality rates determines patterns of above-ground biomass in Amazonian forests: implications for dynamic global vegetation models. *Global Change Biology*, *22*(12), 3996–4013. https://doi.org/https://doi.org/10.1111/gcb.13315

Lewis, S. L., Lopez-Gonzalez, G., Sonké, B., Affum-Baffoe, K., Baker, T. R., Ojo, L. O., Phillips, O. L., Reitsma, J. M., White, L., Comiskey, J. A., Djuikouo K, M. N., Ewango, C. E. N., Feldpausch, T. R., Hamilton, A. C., Gloor, M., Hart, T., Hladik, A., Lloyd, J., Lovett, J. C., … Wöll, H. (2009). Increasing carbon storage in intact African tropical forests. *Nature*, *457*(7232), 1003–1006. https://doi.org/10.1038/nature07771

Metcalf, C. J. E., Clark, J. S., & Clark, D. A. (2009). Tree growth inference and prediction when the point of measurement changes: modelling around buttresses in tropical forests. *Journal of Tropical Ecology*, *25*(1), 1–12. https://doi.org/DOI: 10.1017/S0266467408005646

Phillips, O. L., Malhi, Y., Vinceti, B., Baker, T., Lewis, S. L., Higuchi, N., Laurance, W. F., Vargas, P. N., Martinez, R. V., Laurance, S., Ferreira, L. V, Stern, M., Brown, S., & Grace, J. (2002). CHANGES IN GROWTH OF TROPICAL FORESTS: EVALUATING POTENTIAL BIASES. *Ecological Applications*, *12*(2), 576–587. https://doi.org/https://doi.org/10.1890/1051-0761(2002)012[0576:CIGOTF]2.0.CO;2

Poorter, L., Bongers, L., & Bongers, F. (2006). ARCHITECTURE OF 54 MOIST-FOREST TREE SPECIES: TRAITS, TRADE-OFFS, AND FUNCTIONAL GROUPS. *Ecology*, *87*(5), 1289–1301. https://doi.org/https://doi.org/10.1890/0012-9658(2006)87[1289:AOMTST]2.0.CO;2

Purves, D. W., Lichstein, J. W., & Pacala, S. W. (2007). Crown plasticity and competition for canopy space: A new spatially implicit model parameterized for 250 North American tree species. *PLoS ONE*, *2*(9). https://doi.org/10.1371/journal.pone.0000870

Purves, D. W., Lichstein, J. W., Strigul, N., & Pacala, S. W. (2008). Predicting and understanding forest dynamics using a simple tractable model. *Proceedings of the National Academy of Sciences*, *105*(44), 17018–17022. https://doi.org/10.1073/pnas.0807754105

Quesada, C. A., Phillips, O. L., Schwarz, M., Czimczik, C. I., Baker, T. R., Patiño, S., Fyllas, N. M., Hodnett, M. G., Herrera, R., Almeida, S., Alvarez Dávila, E., Arneth, A., Arroyo, L., Chao, K. J., Dezzeo, N., Erwin, T., di Fiore, A., Higuchi, N., Honorio Coronado, E., … Lloyd, J. (2012). Basin-wide variations in Amazon forest structure and function are mediated by both soils and climate. *Biogeosciences*, *9*(6), 2203–2246. https://doi.org/10.5194/bg-9-2203-2012

Sheil, D. (1995). A critique of permanent plot methods and analysis with examples from Budongo Forest, Uganda. *Forest Ecology and Management*, *77*(1–3), 11–34. https://doi.org/10.1016/0378-1127(95)03583-V

Talbot, J., Lewis, S. L., Lopez-Gonzalez, G., Brienen, R. J. W., Monteagudo, A., Baker, T. R., Feldpausch, T. R., Malhi, Y., Vanderwel, M., Araujo Murakami, A., Arroyo, L. P., Chao, K. J., Erwin, T., van der Heijden, G., Keeling, H., Killeen, T., Neill, D., Núñez Vargas, P., Parada Gutierrez, G. A., … Phillips, O. L. (2014). Methods to estimate aboveground wood productivity from long-term forest inventory plots. *Forest Ecology and Management*, *320*, 30–38. https://doi.org/10.1016/j.foreco.2014.02.021
