# Supplementary material for: Increasing tree size across Amazonia
Source: Nat Plants. 2025 Sep 25;11(10):2016–25. doi: 10.1038/s41477-025-02097-4 (PMC12537487; doi:10.1038/s41477-025-02097-4)
Supplement: Supplementary file 2 — Reporting Summary [file 41477_2025_2097_MOESM2_ESM.pdf]

nature portfolio

Corresponding author(s): Adriane Esquivel Muelbert

Last updated by author(s): Feb 6, 2025

Reporting Summary

Nature Portfolio wishes to improve the reproducibility of the work that we publish. This form provides structure for consistency and transparency in reporting. For further information on Nature Portfolio policies, see our Editorial Policies and the Editorial Policy Checklist.

Statistics

For all statistical analyses, confirm that the following items are present in the figure legend, table legend, main text, or Methods section.

n/a	Confirmed	
☐	☒	The exact sample size (n) for each experimental group/condition, given as a discrete number and unit of measurement
☐	☒	A statement on whether measurements were taken from distinct samples or whether the same sample was measured repeatedly
☐	☒	The statistical test(s) used AND whether they are one- or two-sided<br>*Only common tests should be described solely by name; describe more complex techniques in the Methods section.*
☐	☒	A description of all covariates tested
☐	☒	A description of any assumptions or corrections, such as tests of normality and adjustment for multiple comparisons
☐	☒	A full description of the statistical parameters including central tendency (e.g. means) or other basic estimates (e.g. regression coefficient) AND variation (e.g. standard deviation) or associated estimates of uncertainty (e.g. confidence intervals)
☒	☐	For null hypothesis testing, the test statistic (e.g. F, t, r) with confidence intervals, effect sizes, degrees of freedom and P value noted<br>*Give P values as exact values whenever suitable.*
☒	☐	For Bayesian analysis, information on the choice of priors and Markov chain Monte Carlo settings
☒	☐	For hierarchical and complex designs, identification of the appropriate level for tests and full reporting of outcomes
☒	☐	Estimates of effect sizes (e.g. Cohen's d, Pearson's r), indicating how they were calculated

Our web collection on statistics for biologists contains articles on many of the points above.

Software and code

Policy information about availability of computer code

Data collection	No software was used for data collection.
Data analysis	Forest dynamics data were managed using the ForestPlots.net platform. The analyses were performed using the open-source software R and the packages listed in the main text. All codes have been uploaded to Code Ocean.

For manuscripts utilizing custom algorithms or software that are central to the research but not yet described in published literature, software must be made available to editors and reviewers. We strongly encourage code deposition in a community repository (e.g. GitHub). See the Nature Portfolio guidelines for submitting code & software for further information.

Data

Policy information about availability of data

All manuscripts must include a data availability statement. This statement should provide the following information, where applicable:

- Accession codes, unique identifiers, or web links for publicly available datasets
- A description of any restrictions on data availability
- For clinical datasets or third party data, please ensure that the statement adheres to our policy

Provide your data availability statement here.

Research involving human participants, their data, or biological material

Policy information about studies with human participants or human data. See also policy information about sex, gender (identity/presentation), and sexual orientation and race, ethnicity and racism.

Reporting on sex and gender	NA
Reporting on race, ethnicity, or other socially relevant groupings	NA
Population characteristics	NA
Recruitment	NA
Ethics oversight	NA

Note that full information on the approval of the study protocol must also be provided in the manuscript.

Field-specific reporting

Please select the one below that is the best fit for your research. If you are not sure, read the appropriate sections before making your selection.

☐ Life sciences ☐ Behavioural & social sciences ☒ Ecological, evolutionary & environmental sciences

For a reference copy of the document with all sections, see nature.com/documents/nr-reporting-summary-flat.pdf

Ecological, evolutionary & environmental sciences study design

All studies must disclose on these points even when the disclosure is negative.

Study description	We used permanent forest plots across Amazonia to perform a spatio-temporal analysis of tree size and forest structure across Amazon forests over the last 50 years.
Research sample	We used data from the RAINFOR and PPBio networks accessed via the Forestplots.net repository.
Sampling strategy	Our analyses were performed looking at trends of structure and tree size across Amazonian forests. We used data from existing permanent inventory plots across Amazonia.
Data collection	In every sampling plot, all trees that have stem diameter at 1.3 m (or above buttresses) of ≥10 cm are measured, tagged, and identified, when possible, to the species level. In every census, when the plot is revisited, the living trees are measured, the new recruits that attain stem diameter ≥10 cm are tagged and measured, and notes are taken about the dead trees. Data collection was performed by most of the co-authors with help from hundreds of field assistants.
Timing and spatial scale	We investigated the trends in tree mortality in 188 long-term forest inventory plots measured at least four times between 1970 and 2015 and distributed across Amazonia. The average size of the plots is 1.2 ha (range 0.4 to 12 ha). Plots were monitored on average for 13 years (range 2 to 30 years).
Data exclusions	Lianas and nonwoody arborescent individuals from the families Strelitziaceae and Cyatheaceae were excluded from these analyses.
Reproducibility	Plot monitoring followed a standard protocol that can be find online at: http://www.rainfor.org/en/manuals/in-the-field
Randomization	Allocation of plots vary between different regions of the network and overall aim to capture the landscape observed locally following a stratified random sampling approach. All trees within each plot are monitored.
Blinding	Blinding was not relevant to our study.

Did the study involve field work? ☒ Yes ☐ No

Field work, collection and transport

Field conditions	Temperature in the field varies 25 and 35 degrees Celsius. Field work if often undertaken during the dry season.
Location	All plots were located in lowland (<1,000 m.a.s.l.), terra firme, mature forest. All plots part of RAINFOR permanent inventory plot network and individual location is available upon request.

Access & import/export	We follow the national laws of each individual country and only undertook any field work after having the right permits. This work did not required importing or exporting any material.
Disturbance	Our sampling did not cause significant disturbances on the plot. Botanical vouchers for species identification were collected by canopy climbing with minimum disturbance.

Reporting for specific materials, systems and methods

We require information from authors about some types of materials, experimental systems and methods used in many studies. Here, indicate whether each material, system or method listed is relevant to your study. If you are not sure if a list item applies to your research, read the appropriate section before selecting a response.

Materials & experimental systems

n/a	Involved in the study	
☒	☐	Antibodies
☒	☐	Eukaryotic cell lines
☒	☐	Palaeontology and archaeology
☒	☐	Animals and other organisms
☒	☐	Clinical data
☒	☐	Dual use research of concern
☐	☒	Plants

Methods

n/a	Involved in the study	
☒	☐	ChIP-seq
☒	☐	Flow cytometry
☒	☐	MRI-based neuroimaging

Dual use research of concern

Policy information about dual use research of concern

Hazards

Could the accidental, deliberate or reckless misuse of agents or technologies generated in the work, or the application of information presented in the manuscript, pose a threat to:

No	Yes	
☒	☐	Public health
☒	☐	National security
☒	☐	Crops and/or livestock
☒	☐	Ecosystems
☒	☐	Any other significant area

Experiments of concern

Does the work involve any of these experiments of concern:

No	Yes	
☒	☐	Demonstrate how to render a vaccine ineffective
☒	☐	Confer resistance to therapeutically useful antibiotics or antiviral agents
☒	☐	Enhance the virulence of a pathogen or render a nonpathogen virulent
☒	☐	Increase transmissibility of a pathogen
☒	☐	Alter the host range of a pathogen
☒	☐	Enable evasion of diagnostic/detection modalities
☒	☐	Enable the weaponization of a biological agent or toxin
☒	☐	Any other potentially harmful combination of experiments and agents

Plants

Seed stocks	NA
Novel plant genotypes	NA
Authentication	NA
